# Central Nervous System (CNS) Medication Use Before Suicide Among Older Adults in Sweden From 2007 to 2020: A Register‐Based Case‐Control Study

**DOI:** 10.1002/gps.70235

**Published:** 2026-06-20

**Authors:** Theodore Tianyi Miao, Máté Szilcz, Zheng Chang, Jonas W. Wastesson, Kristina Johnell

**Affiliations:** ^1^ Department of Medical Epidemiology and Biostatistics Karolinska Institutet Stockholm Sweden; ^2^ Aging Research Center Department of Neurobiology Care Sciences and Society Karolinska Institutet and Stockholm University Stockholm Sweden

**Keywords:** CNS medications, drug utilization, epidemiology, older adults, pharmacoepidemiology, suicide

## Abstract

**Background:**

Older adults represent a high‐risk group for suicide and are frequently exposed to central nervous system (CNS) drugs. Yet, the role of CNS drugs in late‐life suicide remains unclear.

**Methods:**

We conducted a nationwide register‐based matched case‐control study (1:30) including all individuals aged 65 years and older who died by suicide in Sweden between 2007 and 2020. Each case was matched to controls from the general population on age and sex, alive at the index date. Use of CNS medications was examined within 1‐month, 3‐month, and 12‐month windows prior to the index date (date of suicide). Conditional logistic regression was used to estimate odds ratios (ORs) with adjustment for sociodemographic factors, number of other classes of medications (proxy for comorbidities), frailty score, self‐harm history and major psychiatric diagnoses.

**Results:**

Among 5971 older adults who died by suicide, 71.7% of cases used at least one type of CNS drug (vs. 35.2% of controls), while 34.7% of cases had dispensations for three or more CNS drug types (vs. 7.3% of controls) within one year before death by suicide. The most common drug classes among cases were hypnotics and sedatives (46.5%) and antidepressants (38.8%). About one third of cases used anxiolytics (32.3%) or minor analgesics and antipyretics (29.9%). Compared with controls, individuals who died by suicide more often used hypnotics and sedatives (adjusted OR 3.54, 95% CI 3.32–3.77), anxiolytics (aOR 3.27, 95% CI 3.04–3.52), antidepressants (aOR 2.50, 95% CI 2.33–2.68), and opioids (aOR 1.93, 95% CI 1.79–2.09) within 12 months before suicide. Patterns were consistent across time windows.

**Conclusions:**

CNS medications are commonly dispensed before suicide in older adults, particularly hypnotics and sedatives, antidepressants, anxiolytics, and opioids. These findings describe medication use patterns preceding suicide and identify drug classes for further investigation.

## Key Points

- CNS medications were commonly used before suicide among older adults, particularly hypnotics/sedatives, antidepressants, anxiolytics, and opioids.
- Older adults who died by suicide had higher use of CNS medications compared with their controls.
- Patterns of CNS drug dispensations were stable during the year before the index date.
- Future studies should further examine how CNS medication use relates to underlying illness, clinical deterioration, and suicide risk in older adults.

## 1 Introduction

Old age is a risk factor for suicide [1]. Globally, individuals aged 85 years and older have the highest suicide rate [2], and they are frequently exposed to central nervous system (CNS) medications [3]. Despite the high suicide rate and high prevalence of CNS medication use in older adults, limited attention has been paid to the role of medications.

Suicide in older adults often occurs in the context of psychiatric disorders and physical illness [1, 4, 5], conditions that contribute to a high overall disease burden [6] and extensive medication use [7, 8, 9]. Older adults commonly use multiple medications, and previous research indicates that older adults who die by suicide are prescribed more medications than the general population [1, 10].

Central nervous system (CNS) medications are widely prescribed in older adults to manage psychiatric disorders, sleep problems, pain, and neurological conditions [3, 4, 11, 12]. Because these drugs act on the brain and are often used in situations involving psychological distress, they are typically present in the clinical context preceding suicide. Describing how all major CNS medication classes are used before suicide may therefore improve the understanding of the treatment context in late‐life suicide and indicate which medication groups warrant closer examination in future studies [13, 14]. Previous research has focused on individual psychotropic medications in relation to suicide or self‐harm, with inconsistent findings and limited attention to older age groups [12, 13, 15, 16]. Few previous studies have described CNS drug use prior to suicide in older adults at the national level [14, 17, 18, 19, 20]. We therefore conducted a nationwide case‐control study covering all major CNS medication classes.

The aim of this study is to describe patterns of real‐world CNS medication use patterns before suicide among Swedish older adults, using a nationwide register‐based case‐control design.

## 2 Method

### 2.1 Data

We analyzed pseudonymized individual‐level data from nationwide Swedish registers (Supporting Information S1: Table S2). Individuals were linked by unique personal identification numbers to obtain data from national covered registries [21]. Demographics data were extracted from the Total Population Register, diagnoses data from the National Patient Register, dispensed prescriptions data from the Swedish Prescribed Drug Register, and deaths (including suicide) data from the Cause of Death Register.

### 2.2 Population

**FIGURE 1:**
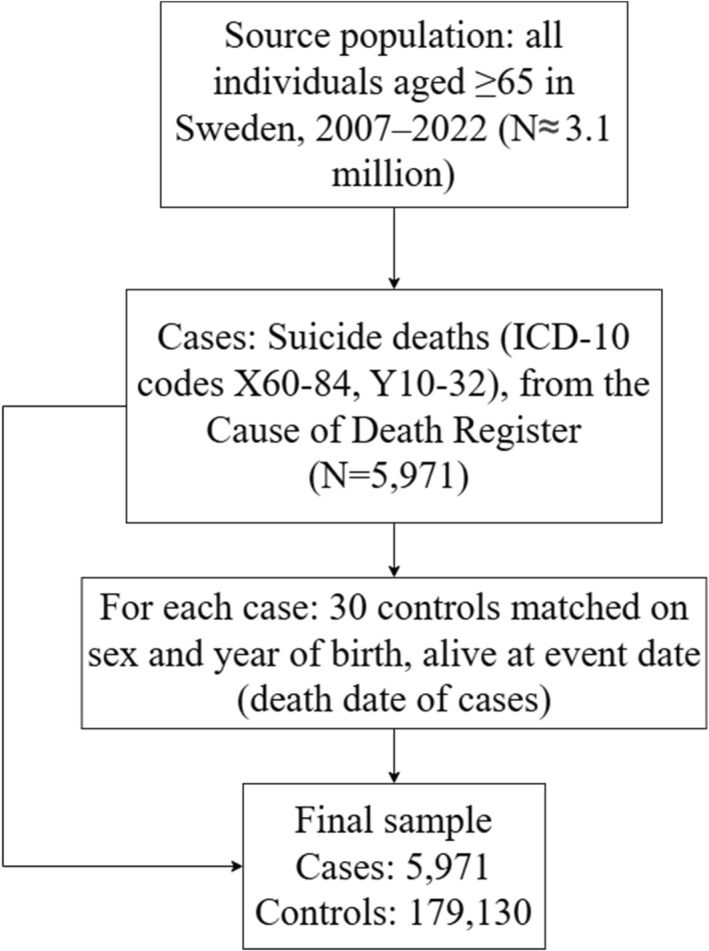
Flowchart of case and control selection.

In the population aged 65 years and older living in Sweden from 2007 to 2020, we included all registered suicide cases in our study (*n* = 5971). Using a matched case‐control design, as depicted in Figure 1, we compared the medication dispensations between older adults who died by suicide (cases) and their control group. For every suicide case, 30 controls (*n* = 179,130) were randomly selected from the general population and matched on sex and year of birth. All controls were required to be alive on the index date, defined as the date of death by suicide of the corresponding case.

### 2.3 Identification of Suicide

In the Swedish Cause of Death Register, the 10th International Classification of Diseases (ICD‐10) codes were used to categorize causes of death [22]. In our study, suicide was identified as deaths with ICD‐10 codes for intentional self‐harm (X60–X84) and events of undetermined intent (Y10–Y32), following common practice in suicide research, to reduce the underestimation of the true suicide cases [23].

### 2.4 Identification of CNS Medication Use

Medication use was assessed through the Swedish Prescribed Drug Register [24], which contains detailed data about all prescription drugs dispensed at Swedish pharmacies. All drugs used by cases and controls were coded using Anatomical Therapeutic Chemical (ATC) codes [25]. Our research team has developed a text parsing algorithm to calculate periods of drug exposure based on the prescribed dosage in free text [26, 27]. In this study, we included the following classes of drugs: antidepressants (ATC code: N06A), antipsychotics (N05A), anxiolytics (N05B), hypnotics and sedatives (N05C), opioids (N02A), antiepileptics (N03A) and minor analgesics and antipyretics (N02B). We did not include psychostimulants (N06B) and anti‐dementia drugs (N06D), because psychostimulants were rarely used among older adults, and anti‐dementia drugs are mostly used to manage dementia and cognitive impairment rather than psychiatric symptoms. Individuals were classified as current users if they had any medication coverage from that CNS class, calculated from dispensing dates, total amount of drugs dispensed, and information from free text, during the 1‐, 3‐, or 12‐month periods preceding the date of death by suicide; otherwise, they were classified as non‐users [25].

### 2.5 Identification of Covariates

Covariates were included to describe demographic and clinical characteristics of the study population and to enable comparison between individuals who died by suicide and matched controls. Covariates included sex, age, civil status, educational level, number of other classes of medications (often used as a proxy for comorbidity [28, 29, 30]), frailty risk score [31], self‐harm history and major psychiatric diagnoses.

Sex was defined as recorded in the Total Population Register, and age was defined as the individual's age in the calendar year of the index date. Civil status and education level were extracted from the Total Population Register and categorized as “married or partnered” (married/registered partner), “single” (unmarried/divorced), and “widowed”. Educational attainment was categorized as primary, secondary, or tertiary. Primary included pre‐secondary education (≤ 9 years); secondary included high school education (2–3 years); and tertiary included post‐secondary or postgraduate education. The proportion of missing data for education and civil status was negligible; nevertheless, missing values were treated as a separate category when modeling.

Self‐harm history is one of the strongest risk factors for suicide [32]. We identified self‐harm history from the National Patient Register, with ICD‐10 codes from X60 to X84 and from Y10 to Y34. Individuals with a recorded self‐harm before index date and after the age of 65 were classified as having a history of self‐harm [1, 3]. Key psychiatric diagnoses within 3 years prior to suicide were defined by ICD‐10 codes from the National Patient Register as follows: substance use disorders (F10–F19), schizophrenia (F20), bipolar disorder (F31), depression (F32–F33), anxiety disorders (F40–F41), and personality disorders (F60–F61) [27].

We used the Swedish Prescribed Drug Register to derive the number of other drug classes (ATC 3rd‐level) other than those we included in main analysis [24]. For consistency, exposure windows for these drug classes were defined using the same time windows as for CNS medications. Individuals were classified as current users if they had any coverage for a given drug class during the 1‐, 3‐, or 12‐month period before the suicide date (based on dispensing date and amount dispensed); otherwise, they were classified as non‐users.

Frailty risk was derived from the National Patient Register using the method described by Gilbert et al. [31] Frailty risk scores lower than 5 was coded as “low frailty risk”, scores between 5 and 10 were coded as “moderate frailty risk” and scores higher than 10 were coded as “high frailty risk”.

### 2.6 Statistical Analysis

Descriptive statistics were used to summarize the characteristics of cases and their matched controls. The case‐control study design was chosen because suicide is a rare event, and this approach allows efficient estimation of associations between time‐varying medication exposure and suicide, an overview of the study design is provided in Supporting Information S1: Figure S1. Conditional logistic regression, suitable for matched data with binary outcomes, was then applied in this study [33]. Each CNS medication class was analyzed separately in different models. Given the matched case‐control design, age and sex were controlled for through matching. In model A, adjustment was made for civil status and education level. In model B, we added the frailty risk score, and the number of other drug classes (i.e., drug classes other than the medication of interest). Model C, the fully adjusted model, further included self‐harm history and diagnoses of major psychiatric disorders, together with all covariates mentioned above. Odds ratios (ORs) with 95% confidence intervals (CIs) were reported. All analyses were conducted using R, version 4.3.3.

To assess the influence of exposure definition on our findings, we conducted a sensitivity analysis in which drug exposure duration was defined based on defined daily dose (DDD) rather than the algorithm‐estimated duration. We calculated the total amount of DDDs dispensed and divided this by the standard DDD to derive the duration of medication exposure.

This study is reported in accordance with the RECORD‐PE (Reporting of studies conducted using observational routinely collected data for pharmacoepidemiology) guideline (see Supporting Information S1: Table S16).

### 2.7 Artificial Intelligence Usage

An artificial intelligence large language model was used to assist with editing and refactoring analysis code (e.g., improving readability and fixing syntactic issues) and to improve grammar and clarity of the manuscript. The authors designed the analyses, executed the code, verified outputs, and take full responsibility for the results and conclusions.

## 3 Results

### 3.1 Patterns of CNS Drug Dispensation

There were 5971 older adults who died by suicide and the majority (69.1%) of them were men (Table 1). About half of the study population were between 65 and 74 years old, and one third was between 75 and 84 years old. Among people who died by suicide, about two‐thirds (65.7%) were not married or partnered (vs. 44.6% in controls), 39.8% had a psychiatric diagnosis before suicide, whereas the prevalence in controls was 6.9%. Within the 1‐year period before suicide, 71.7% of cases used at least one type of CNS drug (vs. 35.2% of controls), while 34.7% of cases used more than three CNS drug types (vs. 7.3% of controls).

**TABLE 1:** Baseline characteristics of suicide and control group.

Characteristics	Number (%) of cases (*n* = 5971)	Number (%) of controls (*n* = 179,130)
Sex		
Men	4125 (69.1)	123,750 (69.1)
Women	1846 (30.9)	55,380 (30.9)
Age (years)		
65–74	3095 (51.8)	92,801 (51.8)
75–84	1916 (32.1)	57,486 (32.1)
85–94	896 (15.0)	26,916 (15.0)
≥ 95	64 (1.1)	1927 (1.1)
Civil status		
Widowed	1252 (21.0)	30,507 (17.0)
Single or divorced	2666 (44.6)	45,875 (25.6)
Married or partnered	2046 (34.3)	99,321 (55.4)
Data not available	7 (0.1)	3427 (1.9)
Education		
Primary	2462 (41.2)	66,474 (37.1)
Secondary	2238 (37.5)	67,540 (37.7)
Tertiary	1146 (19.2)	39,695 (22.2)
Not available	125 (2.1)	5421 (3.0)
Number of dispensed CNS drug classes (1 year before index date)		
0	1692 (28.3)	116,049 (64.8)
1	1073 (18.0)	32,678 (28.2)
2	1132 (19.0)	17,381 (9.7)
≥ 3	2074 (34.7)	13,022 (7.3)
Frailty risk score		
Low frailty risk score	4447 (74.5)	159,786 (89.2)
Moderate frailty risk score	918 (15.4)	12,402 (6.9)
High frailty risk score	606 (10.1)	6942 (3.9)
Self‐harm history	805 (13.5)	1410 (0.8)
Any psychiatric diagnosis^a^	2378 (39.8)	12,335 (6.9)
Depression	1209 (20.2)	2451 (1.4)
Anxiety	728 (12.2)	1691 (0.9)
Bipolar disorder	188 (3.1)	586 (0.3)
Schizophrenia	58 (1)	337 (0.2)
Substance use disorder	838 (14)	2672 (1.5)
Personality disorder	53 (0.9)	87 (0.1)

^a^ Includes the diagnosis of depression, anxiety disorder, bipolar disorder, schizophrenia, substance use disorder, and personality disorder.

**FIGURE 2:**
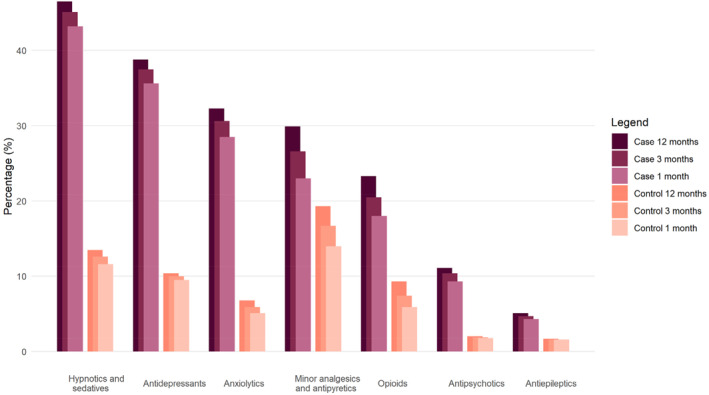
Use of common CNS medications before death by suicide among older adults in Sweden from 2007 to 2020.

Compared with controls, cases used more CNS medication across all drug classes (Figure 2). One year before suicide, the most prevalent CNS medication among cases was hypnotics and sedatives (46.5% vs. 13.5% in controls), followed by antidepressants (38.8% vs. 10% in controls). About one third of cases used anxiolytics (32.3%) and minor analgesics and antipyretics (29.9%), whereas the prevalence in controls was notably lower (6.8% in anxiolytics and 19.3% in minor analgesics and antipyretics). Opioids and antipsychotics were used by 23.3% and 11.1% of cases, compared to 9.3% and 2.0% of controls, respectively. The use of antiepileptics was low in both groups, at 5.1% and 1.7%, respectively. Detailed prevalence tables of CNS drug use before suicide are provided in supplementary materials (Supporting Information S1: Tables S12–S14).

Within the medication classes, prevalences remained largely stable when shifting the time window of medication use from 1 month to 12 months, with prevalence 12 months before suicide about 1%–3% higher than at 3 months and 3%–7% higher than at 1 month.

Table 2 shows the associations between CNS medication use and suicide across the different adjustment models. In the 1‐month period, when medication exposure was analyzed in model A (minimally adjusted model), all CNS medications were associated with increased odds of suicide. After additional adjustment for number of other drug classes and frailty, the OR decreased, particularly for opioids and minor analgesics and antipyretics, which are primarily used for pain management. In the fully adjusted model (Model C), individuals who died by suicide were more likely to use hypnotics and sedatives (OR 3.66, 95% CI 3.43–3.91), antidepressants (OR 2.37, 95% CI 2.20–2.55), anxiolytics (OR 3.58, 95% CI 3.32–3.87). In contrast, the associations for antipsychotics, minor analgesics and antiepileptics were markedly attenuated after full adjustment. Complete model outputs, including estimates for all covariates across the different exposure time windows, are presented in supplementary materials (Supporting Information S1: Tables S3–S11).

**TABLE 2:** Prevalence and adjusted odds ratios of selected cns medication use in the 1‐, 3‐, and 12‐month periods before suicide.

	Case^a^		Control		Model A^b^	Model B^c^	Model C^d^
	*N*	%	*N*	%	OR (95% CI)	OR (95% CI)	OR (95% CI)
One month before suicide							
Hypnotics and sedatives	2580	43.2	20,770	11.6	5.98 (5.66–6.33)	5.12 (4.83–5.44)	3.66 (3.43–3.91)
Antidepressants	2128	35.6	17,047	9.5	5.22 (4.93–5.53)	4.14 (3.89–4.40)	2.37 (2.20–2.55)
Anxiolytics	1700	28.5	9070	5.1	7.11 (6.68–7.57)	5.66 (5.29–6.05)	3.58 (3.32–3.87)
Minor analgesics and antipyretics	1376	23.0	25,163	14.0	1.78 (1.67–1.90)	1.12 (1.04–1.20)	1.08 (1.00–1.17)
Opioids	1076	18.0	10,601	5.9	3.30 (3.08–3.55)	2.24 (2.07–2.41)	2.24 (2.06–2.44)
Antipsychotics	557	9.3	3241	1.8	4.64 (4.22–5.11)	3.32 (3.01–3.67)	1.57 (1.38–1.78)
Antiepileptics	255	4.3	2778	1.6	2.55 (2.24–2.92)	1.63 (1.42–1.87)	1.16 (0.99–1.36)
Three months before suicide							
Hypnotics and sedatives	2691	45.1	22,545	12.6	5.92 (5.60–6.26)	5.00 (4.72–5.31)	3.60 (3.37–3.84)
Antidepressants	2238	37.5	17,950	10.0	5.37 (5.07–5.68)	4.24 (3.99–4.51)	2.44 (2.27–2.62)
Anxiolytics	1827	30.6	10,530	5.9	6.84 (6.43–7.27)	5.39 (5.05–5.76)	3.43 (3.18–3.69)
Minor analgesics and antipyretics	1589	26.6	29,948	16.7	1.77 (1.66–1.88)	1.10 (1.02–1.17)	1.08 (1.00–1.16)
Opioids	1227	20.5	13,246	7.4	3.09 (2.89–3.31)	2.08 (1.93–2.23)	2.07 (1.92–2.24)
Antipsychotics	618	10.4	3428	1.9	4.97 (4.53–5.45)	3.57 (3.25–3.93)	1.72 (1.52–1.94)
Antiepileptics	278	4.7	2938	1.6	2.65 (2.33–3.01)	1.70 (1.49–1.94)	1.20 (1.03–1.40)
Twelve months before suicide							
Hypnotics and sedatives	2776	46.5	24,234	13.5	5.83 (5.51–6.16)	4.90 (4.62–5.20)	3.54 (3.32–3.77)
Antidepressants	2316	38.8	18,714	10.4	5.45 (5.15–5.77)	4.31 (4.05–4.58)	2.50 (2.33–2.68)
Anxiolytics	1929	32.3	12,094	6.8	6.48 (6.11–6.89)	5.11 (4.79–5.45)	3.27 (3.04–3.52)
Minor analgesics and antipyretics	1787	29.9	34,510	19.3	1.77 (1.66–1.87)	1.09 (1.02–1.16)	1.07 (1.00–1.15)
Opioids	1391	23.3	16,674	9.3	2.87 (2.69–3.06)	1.92 (1.80–2.06)	1.93 (1.79–2.09)
Antipsychotics	662	11.1	3599	2.0	5.13 (4.70–5.61)	3.68 (3.35–4.03)	1.76 (1.56–1.98)
Antiepileptics	302	5.1	3086	1.7	2.76 (2.44–3.12)	1.78 (1.57–2.02)	1.23 (1.06–1.43)

^a^ Older adults who died by suicide.

^b^ Adjusted for education level, and civil status.

^c^ Adjusted for education level, civil status, number of other drugs (proxy for comorbidities), and frailty (measured using the Hospital Frailty Risk Score) [31].

^d^ Adjusted for education level, civil status, number of other drugs (proxy for comorbidities), frailty, psychiatric disorders, and self‐harm history.

In the sensitivity analysis, when drug exposure duration was defined according to DDD instead of the algorithm‐based estimate, the results remained largely unchanged (Supporting Information S1: Table S15).

## 4 Discussion

In this nationwide register‐based case‐control study, older adults who died by suicide had two to five times higher odds of CNS medication use than matched population controls. After adjustment for covariates, the difference in CNS medication use varied across classes, but remained higher in cases than controls. CNS medication use patterns were similar across the 1‐, 3‐ and 12‐month windows, suggesting that these patterns reflect longer‐term treatment trajectories rather than short‐term changes immediately preceding suicide.

Our findings align with previous research showing that a higher proportion of older adults who died by suicide used hypnotics and sedatives, antidepressants, and antipsychotics compared to controls [17, 34]. The prevalence of antidepressants, antipsychotics, anxiolytics and opioids was similar to that reported in an earlier Swedish study of older adults who died by suicide [35]. However, the prevalence of these medications in our study was lower than reported in a comparable Norwegian study [15]. For example, in our study approximately 40% of individuals used antidepressants within 12 months prior to death by suicide, whereas the corresponding prevalence in the Norwegian study was 51%. This may reflect differences in mental health disorder management in the two countries.

The higher odds of CNS medication use among individuals who died by suicide compared with matched controls likely reflects the greater burden of both physical and mental health disorders in this group [1, 4, 5]. Similar findings have been reported in previous studies in Sweden [12].

As expected, after adjustment for frailty risk score and number of other classes of drugs (proxy for comorbidity), the differences in the use of minor analgesics and antipyretics and opioids decreased substantially, consistent with their primary use in pain management. Further adjustment for psychiatric diagnoses and self‐harm history largely attenuated the differences in the use of primarily psychotropic medications. This suggests that a large part of the difference in psychotropics came from confounding by indication, whereby the higher prevalence of psychotropic medication use among cases is likely driven by the underlying psychiatric conditions, rather than the medications themselves. However, psychiatric diagnoses captured in the National Patient Register are unlikely to fully reflect underlying psychiatric morbidity. It has been estimated that, among older adults, 50% of less severe psychiatric conditions are diagnosed in primary care and therefore not captured in the register [36]. If these conditions had been fully captured, the observed associations might have been attenuated even further.

The differences for minor analgesics and opioids remained unchanged after adjustment for psychiatric diagnosis and self‐harm history. Minor analgesics and antipyretics are also available over the counter, meaning that their use is not entirely captured in the Swedish Prescribed Drug Register, and may therefore be underestimated in both cases and controls. In addition, the difference for these medications was already small before adjustment for psychiatric diagnoses, suggesting that their relationship with suicide in later life may be limited. Opioids are also highly regulated medications, and are generally prescribed cautiously, particularly in older populations and individuals with known psychiatric disorders or histories of self‐harm [12].

Finally, the similar prevalence across the 1‐, 3‐, and 12‐month windows indicate stable patterns of CNS drug dispensations during the year before the index date, consistent with the chronic nature of CNS treatment in older adults [37].

### 4.1 Implications

This study provides insights into patterns of CNS medication use prior to suicide in the older population in Sweden. Traditional approaches to identifying individuals at high risk of suicide primarily focus on severe mental health disorders. Our findings suggest that, even after adjustment, CNS medication use was more likely among cases than controls, although use was common in both groups. These findings may help characterize individuals with complex clinical needs, including those with psychiatric symptoms, sleep problems, pain, or other conditions requiring CNS medication. However, they should not be interpreted as evidence that CNS medication use alone can identify individuals at high risk of suicide. Further research is needed to examine more direct associations between specific CNS medication classes and suicide.

The patterns observed indicate that suicide in later life frequently occurs in the context of complex treatment patterns involving multiple CNS medications commonly prescribed in routine care. These treatment patterns likely reflect complex clinical needs, including psychological distress, sleep problems, pain, and multimorbidity. Further research is needed to better understand how these treatment trajectories relate to suicide risk in older adults.

These findings should also be interpreted within the Swedish clinical context. CNS medication use before suicide may differ across countries with different health care systems, prescribing practices and levels of primary care and psychiatric service coverage. The patterns observed are best viewed as signals of clinical complexity and treatment trajectories in Sweden.

Future research should examine the temporal patterns of CNS medication use. In our study, CNS medication use was assessed within retrospective exposure windows of 1, 3, and 12 months before suicide. Although this approach captured recent medication use, it did not distinguish between new and chronic users or account for switching and discontinuation. Methods such as self‐controlled case series, case‐crossover designs, or trajectory‐ and clustering‐based approaches may help clarify how changes in CNS medication used over time relate to suicide risk among older adults. Particular attention should be given to antidepressants, hypnotics/sedatives, anxiolytics, and opioids, as these classes were more commonly used among individuals who died by suicide than among matched controls.

### 4.2 Strengths and Limitations

This study has several strengths, including its nationwide coverage, complete ascertainment of suicides, and the use of large high‐quality Swedish register data on dispensed CNS medications.

Some limitations should be noted. The analysis was based on dispensed prescriptions, and we could not account for medications that were prescribed but not filled or other forms of non‐adherence. Thus, our analyses are based on the assumption that individuals who filled their prescriptions adhered to the medication regimen as prescribed. When defining the drug exposure window, we did not apply a grace period or allow stockpiling because this study did not aim to reconstruct exact treatment periods. Instead, we provide a descriptive overview of dispensing patterns across CNS drug classes in this population. In addition, information on over‐the‐counter (OTC) medication use was not available, which may have led to an underestimation of use of drug classes commonly obtained without prescription (e.g., minor analgesics/antipyretics) in both cases and controls.

Information on psychiatric diagnoses and other diagnoses was obtained from hospital and specialized outpatient care, meaning that conditions managed only in primary care were not captured [36]. Therefore, our diagnoses data underestimated the overall disease burden. Moreover, data on disease severity were not available, which limits interpretation of whether observed patterns reflect underlying illness or treatment practices.

This study has limitations related to data availability. Because our data were available from 2007 to 2020, the study period did not fully capture the COVID‐19 pandemic.

In addition, information on race or ethnicity was not available in our data. The generalizability of our findings may be limited, particularly where health care system factors, healthcare help‐seeking behavior, prescribing norms and medication adherence differ from those in Sweden.

### 4.3 Conclusions

CNS medications are commonly used before suicide among older adults, particularly hypnotics, sedatives, antidepressants, anxiolytics, and opioids. Our findings may help identify older adults at high risk and highlight the most important medication classes for further studies. Future research is needed to better understand how CNS medications relate to underlying illness and suicide risk in older adults, with particular attention to temporal patterns of medication use.

## Author Contributions

K.J., J.W.W., M.S., T.T.M. conceived and designed the study. T.T.M. performed statistical analysis, interpreted the data, drafted and critically revised the manuscript. K.J., J.W.W., M.S., and Z.C. interpreted the data and critically revised the manuscript. K.J. obtained funding and acquired the data. K.J., J.W.W., and M.S. provided supervision. K.J., and T.T.M. are the guarantors of study and data integrity. All authors approved the final version of the manuscript.

## Funding

This work was supported by funding from the Swedish Research Council for Health, Working Life and Welfare (FORTE) (Grant Nos. 2023‐00144, 2023‐00164). Sponsor's Role: The funders had no role in the study design, data collection and analysis, decision to publish, or preparation of the manuscript.

## Consent

Consent was not necessary for the present register‐based study. The study was approved by the Regional Ethical Review Board in Stockholm [dnr: 2016/1001–31/4, 2020–03525; 2021–02004].

## Conflicts of Interest

Z.C. received lecture honoraria from Takeda Pharmaceuticals, outside the submitted work.

## Supporting information


Supporting Information S1

## Data Availability

Data may be obtained from a third party and are not publicly available. Personal data cannot be made publicly available because of privacy issues.
