# Supplementary material for: Central Nervous System (CNS) Medication Use Before Suicide Among Older Adults in Sweden From 2007 to 2020: A Register‐Based Case‐Control Study
Source: Int J Geriatr Psychiatry. 2026 Jun 20;41(6):e70235. doi: 10.1002/gps.70235 (PMC13283041; doi:10.1002/gps.70235)
Supplement: Supplementary file 1 — Supporting Information S1 [file GPS-41-e70235-s001.docx]

Central Nervous System (CNS) Medication Use Before Suicide Among Older Adults in Sweden from 2007 to 2020: a register-based case control study

**Supplementary materials**

##

***Supplementary Figure S1: Schematic study design***


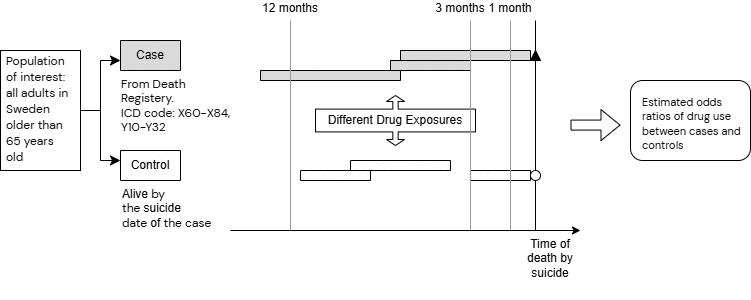


Supplementary Figure S1 shows a schematic study design using two hypothetical individuals. The upper timeline represents a case, for whom the black triangle indicates the date of death by suicide. The lower timeline represents the matched control. The horizontal bars indicate periods of treatment with CNS medications.

***Supplementary Table S1: Data sources***

| **Register** | **Register holder** | **Start** | **Completeness** | **Geographical coverage** | **Content** | **Externally validated** |
| --- | --- | --- | --- | --- | --- | --- |
| Total Population Register^1^ | Statistics Sweden | 1968 | The over-coverage has been estimated to 0.1 % for Nordic citizens but substantially higher for individuals born outside the Nordic countries (potentially 4–8 %) | Nationwide | Sex, date of birth, country of birth, municipality of residence, living arrangement, civil status, year of first and last immigration, year of last emigration | Yes |
| National Cause Of Death Register^2^ | National Board of Health and Welfare | Complete since 1952 | 99% of all deaths are documented with an underlying cause of death | Nationwide | Sex, date of birth, underlying and contributing causes of death (ICD-10), place of death, civil status | Yes |
| National Patient Register^3^ | National Board of Health and Welfare | Inpatient care coverage complete since 1987  Specialised outpatient care since 2001 | 99% of all somatic and psychiatric hospital discharges are registered.  Outpatient diagnoses coverage lower than inpatient data but above 80% | Nationwide | Sex, age, date of admission and discharge, planned/unplanned admission, main diagnosis, secondary diagnoses, injuries, medical or surgical procedures, department of admission, provenance, destination. | The inpatient register part has an overall positive predictive value of diagnosis of 85%-95% |
| National Prescribed Drug Register^4,5^ | National Board of Health and Welfare | July 2005 | 85% of all sold defined daily doses (DDDs) are covered by the register  The remaining 15% are Over-the-counter medications (12% of DDDs), drugs administered in hospitals (3% of DDDs) | Nationwide | Sex, age, date of prescription, date of dispensing, type of dispensing (e.g. multi-dose), total dose, prescribed daily dose (free text), number of DDDs dispensed, ATC code, generic name, costs, characteristics of prescribers | Yes |
| Swedish Register of Education^6^ |  | 1985 (annual updates from 2000), earlier versions have been produced in the 1930 and 1970 census | Population aged 16-74 years old registered as resident in Sweden at 1 January each year. From 2007, information for the group 75+ is also collected | Nationwide | Highest educational attainment | Yes |

|  |  |  |  |  |  |  |  |  |
| --- | --- | --- | --- | --- | --- | --- | --- | --- |
| Drug |  | Opioids | Minor analgesics and antipyretics | Antiepileptics | Antipsychotics | Anxiolytics | Hypnotics and sedatives | Antidepressants |
|  |  | 2.24 (2.06–2.44) | 1.08 (1.00–1.17) | 1.16 (0.99–1.36) | 1.57 (1.38–1.78) | 3.58 (3.32–3.87) | 3.66 (3.43–3.91) | 2.37 (2.20–2.55) |
| Education | Primary | Ref | Ref | Ref | Ref | Ref | Ref | Ref |
|  | Secondary | 0.91 (0.85–0.97) | 0.90 (0.85–0.96) | 0.90 (0.85–0.96) | 0.90 (0.85–0.96) | 0.91 (0.85–0.97) | 0.88 (0.82–0.94) | 0.90 (0.85–0.96) |
|  | Tertiary | 0.85 (0.78–0.92) | 0.84 (0.78–0.91) | 0.84 (0.78–0.91) | 0.84 (0.78–0.91) | 0.85 (0.78–0.92) | 0.80 (0.74–0.87) | 0.84 (0.77–0.91) |
|  | Not available | 0.88 (0.72–1.07) | 0.89 (0.73–1.09) | 0.89 (0.73–1.09) | 0.89 (0.73–1.09) | 0.89 (0.73–1.08) | 0.88 (0.72–1.08) | 0.91 (0.75–1.11) |
| Civil Status | Married or Partnered | Ref | Ref | Ref | Ref | Ref | Ref | Ref |
|  | Single or Divorced | 2.37 (2.23–2.53) | 2.41 (2.26–2.57) | 2.41 (2.26–2.57) | 2.39 (2.25–2.55) | 2.33 (2.18–2.48) | 2.32 (2.17–2.47) | 2.39 (2.24–2.55) |
|  | Widow | 1.83 (1.68–1.99) | 1.84 (1.69–2.00) | 1.84 (1.69–2.00) | 1.83 (1.69–2.00) | 1.82 (1.67–1.98) | 1.79 (1.64–1.94) | 1.79 (1.65–1.95) |
|  | Data not available | 0.21 (0.11–0.39) | 0.21 (0.11–0.40) | 0.21 (0.11–0.40) | 0.21 (0.11–0.40) | 0.20 (0.10–0.38) | 0.21 (0.11–0.39) | 0.21 (0.11–0.40) |
| Frailty Risk Score | Low Frailty Risk | Ref | Ref | Ref | Ref | Ref | Ref | Ref |
|  | Medium Frailty Risk | 1.34 (1.23–1.46) | 1.37 (1.25–1.49) | 1.37 (1.25–1.49) | 1.37 (1.25–1.49) | 1.36 (1.25–1.49) | 1.37 (1.25–1.50) | 1.32 (1.21–1.44) |
|  | High Frailty Risk | 1.06 (0.95–1.18) | 1.12 (1.00–1.25) | 1.12 (1.00–1.25) | 1.11 (0.99–1.24) | 1.12 (1.00–1.25) | 1.14 (1.02–1.27) | 1.06 (0.95–1.18) |
| Number of Other Classes of Drugs |  | 1.04 (1.03–1.05) | 1.06 (1.05–1.07) | 1.06 (1.05–1.07) | 1.06 (1.05–1.07) | 1.03 (1.02–1.03) | 1.01 (1.00–1.02) | 1.04 (1.03–1.04) |
| Self-harm History | Yes | 7.13 (6.35–8.01) | 7.22 (6.43–8.10) | 7.21 (6.43–8.10) | 7.21 (6.42–8.09) | 7.02 (6.24–7.89) | 6.64 (5.90–7.47) | 6.96 (6.19–7.81) |
| Psychiatric diagnosis | Depression | 6.55 (5.95–7.21) | 6.40 (5.82–7.05) | 6.40 (5.81–7.04) | 6.16 (5.59–6.78) | 5.55 (5.04–6.12) | 5.32 (4.83–5.87) | 4.53 (4.10–5.00) |
|  | Anxiety | 3.09 (2.73–3.50) | 3.07 (2.71–3.47) | 3.07 (2.71–3.47) | 3.00 (2.65–3.39) | 2.11 (1.86–2.39) | 2.56 (2.26–2.91) | 2.59 (2.29–2.92) |
|  | Bipolar Disorder | 3.33 (2.69–4.12) | 3.14 (2.54–3.89) | 3.07 (2.47–3.81) | 2.55 (2.04–3.18) | 2.73 (2.20–3.39) | 2.58 (2.08–3.20) | 2.73 (2.20–3.37) |
|  | Schizophrenia | 2.46 (1.78–3.39) | 2.31 (1.68–3.19) | 2.31 (1.68–3.18) | 1.74 (1.25–2.43) | 1.92 (1.38–2.65) | 1.95 (1.41–2.71) | 2.19 (1.59–3.02) |
|  | Substance Use Disorder | 3.40 (3.05–3.79) | 3.48 (3.12–3.87) | 3.48 (3.12–3.87) | 3.48 (3.13–3.88) | 3.30 (2.96–3.69) | 3.07 (2.75–3.42) | 3.47 (3.12–3.87) |
|  | Personality Disorder | 1.73 (1.08–2.78) | 1.71 (1.07–2.74) | 1.71 (1.06–2.73) | 1.57 (0.99–2.51) | 1.43 (0.88–2.32) | 1.38 (0.86–2.23) | 1.74 (1.09–2.76) |
|  |  |  |  |  |  |  |  |  |

***Supplementary Table S2: Adjusted Odds Ratios of Selected CNS Medication Use in the One Month Period Before Death by Suicide***

Complete fully adjusted model outputs for CNS medications separately, including estimates for all covariates one month before death by suicide.

***Supplementary Table S3: Adjusted Odds Ratios of Selected CNS Medication Use in the Three Months Period Before Death by Suicide***

|  |  |  |  |  |  |  |  |  |
| --- | --- | --- | --- | --- | --- | --- | --- | --- |
|  |  | Opioids | Minor analgesics and antipyretics | Antiepileptics | Antipsychotics | Anxiolytics | Hypnotics and sedatives | Antidepressants |
|  |  | 2.07 (1.92–2.24) | 1.08 (1.00–1.16) | 1.20 (1.03–1.40) | 1.72 (1.52–1.94) | 3.43 (3.18–3.69) | 3.60 (3.37–3.84) | 2.44 (2.27–2.62) |
| Education | Primary | Ref | Ref | Ref | Ref | Ref | Ref | Ref |
|  | Secondary | 0.90 (0.85–0.96) | 0.90 (0.85–0.96) | 0.90 (0.85–0.96) | 0.90 (0.85–0.96) | 0.91 (0.85–0.97) | 0.88 (0.82–0.94) | 0.90 (0.85–0.96) |
|  | Tertiary | 0.84 (0.78–0.91) | 0.84 (0.77–0.91) | 0.84 (0.77–0.91) | 0.84 (0.78–0.91) | 0.85 (0.78–0.92) | 0.80 (0.74–0.86) | 0.84 (0.77–0.91) |
|  | Not available | 0.88 (0.72–1.07) | 0.89 (0.73–1.09) | 0.89 (0.73–1.09) | 0.89 (0.73–1.08) | 0.90 (0.73–1.09) | 0.89 (0.73–1.08) | 0.91 (0.75–1.11) |
| Civil Status | Married or Partnered | Ref | Ref | Ref | Ref | Ref | Ref | Ref |
|  | Single or Divorced | 2.38 (2.23–2.54) | 2.41 (2.26–2.57) | 2.41 (2.26–2.57) | 2.39 (2.24–2.55) | 2.33 (2.18–2.48) | 2.31 (2.17–2.47) | 2.39 (2.24–2.54) |
|  | Widow | 1.83 (1.68–1.99) | 1.84 (1.69–2.00) | 1.84 (1.69–2.00) | 1.84 (1.69–2.00) | 1.81 (1.66–1.97) | 1.78 (1.64–1.94) | 1.79 (1.65–1.95) |
|  | Data not available | 0.21 (0.11–0.40) | 0.22 (0.12–0.42) | 0.22 (0.12–0.42) | 0.22 (0.12–0.41) | 0.20 (0.11–0.39) | 0.21 (0.11–0.41) | 0.22 (0.12–0.42) |
| Frailty Risk Score | Low Frailty Risk | Ref | Ref | Ref | Ref | Ref | Ref | Ref |
|  | Medium Frailty Risk | 1.32 (1.21–1.44) | 1.34 (1.23–1.47) | 1.34 (1.23–1.46) | 1.34 (1.23–1.47) | 1.34 (1.22–1.46) | 1.36 (1.24–1.48) | 1.29 (1.18–1.41) |
|  | High Frailty Risk | 1.04 (0.93–1.16) | 1.10 (0.98–1.23) | 1.09 (0.98–1.22) | 1.08 (0.97–1.21) | 1.10 (0.99–1.23) | 1.12 (1.00–1.25) | 1.04 (0.93–1.16) |
| Number of Other Classes of Drugs |  | 1.04 (1.03–1.05) | 1.06 (1.05–1.07) | 1.06 (1.05–1.07) | 1.06 (1.05–1.07) | 1.03 (1.02–1.04) | 1.01 (1.00–1.02) | 1.04 (1.03–1.04) |
| Self-harm History | Yes | 7.10 (6.32–7.97) | 7.20 (6.41–8.08) | 7.19 (6.41–8.08) | 7.19 (6.40–8.07) | 6.99 (6.22–7.86) | 6.61 (5.87–7.45) | 6.92 (6.16–7.77) |
| Psychiatric diagnosis | Depression | 6.48 (5.89–7.14) | 6.37 (5.79–7.01) | 6.36 (5.78–6.99) | 6.05 (5.49–6.66) | 5.43 (4.92–5.98) | 5.28 (4.79–5.82) | 4.41 (4.00–4.87) |
|  | Anxiety | 3.08 (2.72–3.48) | 3.04 (2.68–3.43) | 3.03 (2.68–3.43) | 2.94 (2.59–3.32) | 2.09 (1.85–2.38) | 2.53 (2.24–2.87) | 2.52 (2.23–2.85) |
|  | Bipolar Disorder | 3.26 (2.63–4.04) | 3.11 (2.51–3.85) | 3.01 (2.42–3.74) | 2.37 (1.90–2.96) | 2.70 (2.17–3.35) | 2.52 (2.03–3.13) | 2.68 (2.17–3.32) |
|  | Schizophrenia | 2.44 (1.77–3.36) | 2.30 (1.67–3.17) | 2.29 (1.67–3.16) | 1.62 (1.16–2.25) | 1.92 (1.39–2.66) | 1.94 (1.40–2.70) | 2.16 (1.56–2.99) |
|  | Substance Use Disorder | 3.37 (3.02–3.75) | 3.45 (3.10–3.85) | 3.45 (3.10–3.85) | 3.46 (3.11–3.85) | 3.30 (2.96–3.68) | 3.05 (2.73–3.40) | 3.43 (3.08–3.81) |
|  | Personality Disorder | 1.74 (1.09–2.80) | 1.69 (1.05–2.70) | 1.68 (1.05–2.69) | 1.50 (0.94–2.39) | 1.42 (0.88–2.30) | 1.40 (0.87–2.26) | 1.68 (1.06–2.66) |

Complete fully adjusted model outputs for CNS medications separately, including estimates for all covariates three months before death by suicide.

***Supplementary Table S4: Adjusted Odds Ratios of Selected CNS Medication Use in the One Year Period Before Death by Suicide***

|  |  | Opioids | Minor analgesics and antipyretics | Antiepileptics | Antipsychotics | Anxiolytics | Hypnotics and sedatives | Antidepressants |
| --- | --- | --- | --- | --- | --- | --- | --- | --- |
|  |  | 1.93 (1.79–2.09) | 1.07 (1.00–1.15) | 1.23 (1.06–1.43) | 1.76 (1.56–1.98) | 3.27 (3.04–3.52) | 3.54 (3.32–3.77) | 2.50 (2.33–2.68) |
| Education | Primary | Ref | Ref | Ref | Ref | Ref | Ref | Ref |
|  | Secondary | 0.90 (0.85–0.96) | 0.90 (0.84–0.96) | 0.90 (0.84–0.96) | 0.90 (0.85–0.96) | 0.91 (0.85–0.97) | 0.88 (0.82–0.94) | 0.90 (0.85–0.96) |
|  | Tertiary | 0.84 (0.78–0.91) | 0.83 (0.77–0.90) | 0.83 (0.77–0.90) | 0.84 (0.77–0.91) | 0.84 (0.78–0.91) | 0.79 (0.73–0.86) | 0.83 (0.77–0.90) |
|  | Not available | 0.87 (0.72–1.07) | 0.89 (0.73–1.08) | 0.89 (0.73–1.08) | 0.89 (0.73–1.08) | 0.89 (0.73–1.09) | 0.88 (0.72–1.08) | 0.91 (0.75–1.11) |
| Civil Status | Married or Partnered | Ref | Ref | Ref | Ref | Ref | Ref | Ref |
|  | Single or Divorced | 2.39 (2.24–2.54) | 2.41 (2.26–2.57) | 2.41 (2.26–2.57) | 2.39 (2.24–2.55) | 2.33 (2.18–2.49) | 2.31 (2.17–2.47) | 2.39 (2.24–2.54) |
|  | Widow | 1.83 (1.68–1.99) | 1.84 (1.69–2.00) | 1.84 (1.69–2.00) | 1.84 (1.69–2.00) | 1.80 (1.66–1.96) | 1.78 (1.63–1.93) | 1.79 (1.65–1.95) |
|  | Data not available | 0.21 (0.11–0.41) | 0.22 (0.12–0.42) | 0.22 (0.12–0.42) | 0.22 (0.12–0.42) | 0.21 (0.11–0.39) | 0.22 (0.11–0.41) | 0.22 (0.12–0.42) |
| Frailty Risk Score | Low Frailty Risk | Ref | Ref | Ref | Ref | Ref | Ref | Ref |
|  | Medium Frailty Risk | 1.31 (1.20–1.43) | 1.33 (1.22–1.46) | 1.33 (1.22–1.45) | 1.33 (1.22–1.45) | 1.33 (1.21–1.45) | 1.35 (1.23–1.47) | 1.28 (1.17–1.40) |
|  | High Frailty Risk | 1.03 (0.92–1.15) | 1.09 (0.97–1.21) | 1.08 (0.96–1.20) | 1.07 (0.96–1.19) | 1.08 (0.97–1.21) | 1.10 (0.99–1.23) | 1.02 (0.92–1.14) |
| Number of Other Classes of Drugs |  | 1.04 (1.03–1.05) | 1.06 (1.05–1.07) | 1.06 (1.05–1.07) | 1.06 (1.05–1.06) | 1.03 (1.02–1.03) | 1.01 (1.01–1.02) | 1.03 (1.03–1.04) |
| Self-harm History | Yes | 7.12 (6.34–8.00) | 7.20 (6.42–8.08) | 7.20 (6.41–8.08) | 7.19 (6.41–8.07) | 6.98 (6.20–7.85) | 6.58 (5.85–7.41) | 6.91 (6.15–7.75) |
| Psychiatric diagnosis | Depression | 6.49 (5.89–7.14) | 6.37 (5.79–7.01) | 6.35 (5.77–6.99) | 5.99 (5.44–6.60) | 5.39 (4.89–5.94) | 5.27 (4.78–5.80) | 4.34 (3.93–4.79) |
|  | Anxiety | 3.06 (2.71–3.47) | 3.02 (2.67–3.42) | 3.02 (2.67–3.41) | 2.91 (2.57–3.29) | 2.11 (1.86–2.39) | 2.53 (2.23–2.86) | 2.49 (2.21–2.82) |
|  | Bipolar Disorder | 3.25 (2.62–4.02) | 3.11 (2.51–3.85) | 2.98 (2.40–3.71) | 2.33 (1.86–2.90) | 2.71 (2.18–3.36) | 2.53 (2.04–3.14) | 2.67 (2.15–3.30) |
|  | Schizophrenia | 2.46 (1.79–3.40) | 2.32 (1.68–3.19) | 2.31 (1.67–3.18) | 1.59 (1.14–2.22) | 1.94 (1.41–2.69) | 1.94 (1.40–2.70) | 2.18 (1.57–3.01) |
|  | Substance Use Disorder | 3.37 (3.02–3.75) | 3.44 (3.09–3.83) | 3.44 (3.09–3.83) | 3.44 (3.09–3.84) | 3.30 (2.95–3.68) | 3.06 (2.74–3.41) | 3.39 (3.04–3.77) |
|  | Personality Disorder | 1.72 (1.07–2.76) | 1.68 (1.05–2.70) | 1.67 (1.04–2.68) | 1.47 (0.92–2.34) | 1.39 (0.86–2.24) | 1.44 (0.89–2.33) | 1.67 (1.06–2.65) |

Complete fully adjusted model outputs for CNS medications separately, including estimates for all covariates one year before death by suicide.

***Supplementary Table S5: Adjusted Odds Ratios of Selected CNS Medication Use for Women in the One Month Period Before Death by Suicide***

|  |  |  |  |  |  |  |  |  |
| --- | --- | --- | --- | --- | --- | --- | --- | --- |
|  |  | Opioids | Minor analgesics and antipyretics | Antiepileptics | Antipsychotics | Anxiolytics | Hypnotics and sedatives | Antidepressants |
|  |  | 2.01 (1.73–2.33) | 0.94 (0.82–1.08) | 1.03 (0.76–1.40) | 1.95 (1.58–2.39) | 3.55 (3.11–4.04) | 3.75 (3.33–4.22) | 2.20 (1.94–2.50) |
| Education | Primary | Ref | Ref | Ref | Ref | Ref | Ref | Ref |
|  | Secondary | 1.02 (0.90–1.16) | 1.02 (0.90–1.16) | 1.03 (0.90–1.16) | 1.03 (0.91–1.17) | 1.03 (0.91–1.17) | 0.98 (0.87–1.12) | 1.04 (0.91–1.18) |
|  | Tertiary | 1.16 (1.00–1.35) | 1.15 (1.00–1.34) | 1.16 (1.00–1.34) | 1.17 (1.01–1.36) | 1.20 (1.04–1.40) | 1.10 (0.94–1.27) | 1.16 (1.00–1.35) |
|  | Not available | 1.26 (0.89–1.77) | 1.28 (0.91–1.81) | 1.27 (0.90–1.79) | 1.28 (0.91–1.80) | 1.34 (0.95–1.89) | 1.33 (0.93–1.88) | 1.31 (0.93–1.85) |
| Civil Status | Married or Partnered | Ref | Ref | Ref | Ref | Ref | Ref | Ref |
|  | Single or Divorced | 2.33 (2.04–2.66) | 2.38 (2.08–2.71) | 2.37 (2.08–2.70) | 2.34 (2.06–2.67) | 2.28 (1.99–2.60) | 2.29 (2.00–2.61) | 2.35 (2.06–2.68) |
|  | Widow | 1.59 (1.37–1.85) | 1.60 (1.38–1.86) | 1.59 (1.37–1.85) | 1.59 (1.37–1.84) | 1.56 (1.34–1.82) | 1.55 (1.33–1.80) | 1.55 (1.34–1.81) |
|  | Data not available | 0.07 (0.01–0.58) | 0.07 (0.01–0.60) | 0.07 (0.01–0.59) | 0.07 (0.01–0.60) | 0.06 (0.01–0.52) | 0.07 (0.01–0.60) | 0.09 (0.01–0.66) |
| Frailty Risk Score | Low Frailty Risk | Ref | Ref | Ref | Ref | Ref | Ref | Ref |
|  | Medium Frailty Risk | 1.41 (1.20–1.66) | 1.46 (1.24–1.71) | 1.45 (1.23–1.70) | 1.46 (1.24–1.72) | 1.43 (1.21–1.69) | 1.49 (1.26–1.75) | 1.42 (1.21–1.67) |
|  | High Frailty Risk | 1.23 (1.01–1.51) | 1.33 (1.09–1.63) | 1.32 (1.08–1.61) | 1.30 (1.06–1.59) | 1.34 (1.10–1.64) | 1.48 (1.21–1.81) | 1.28 (1.05–1.56) |
| Number of Other Classes of Drugs |  | 1.04 (1.03–1.06) | 1.07 (1.06–1.09) | 1.07 (1.05–1.08) | 1.06 (1.05–1.08) | 1.03 (1.01–1.04) | 1.01 (0.99–1.02) | 1.04 (1.02–1.05) |
| Self-harm History | Yes | 11.82 (9.74–14.34) | 12.06 (9.95–14.61) | 12.06 (9.95–14.62) | 12.11 (9.99–14.68) | 11.49 (9.44–13.99) | 11.09 (9.11–13.51) | 11.39 (9.39–13.81) |
| Psychaitric diagnosis | Depression | 6.47 (5.50–7.61) | 6.28 (5.35–7.38) | 6.29 (5.36–7.40) | 5.96 (5.07–7.01) | 5.60 (4.76–6.60) | 5.24 (4.45–6.18) | 4.78 (4.05–5.64) |
|  | Anxiety | 3.50 (2.89–4.22) | 3.48 (2.88–4.20) | 3.48 (2.88–4.20) | 3.34 (2.77–4.04) | 2.33 (1.92–2.84) | 2.86 (2.36–3.47) | 2.94 (2.44–3.55) |
|  | Bipolar Disorder | 3.45 (2.43–4.90) | 3.21 (2.27–4.55) | 3.28 (2.29–4.69) | 2.34 (1.62–3.36) | 2.79 (1.95–3.99) | 2.64 (1.85–3.78) | 2.82 (1.99–4.00) |
|  | Schizophrenia | 4.53 (2.72–7.55) | 4.38 (2.63–7.30) | 4.35 (2.61–7.25) | 2.78 (1.63–4.75) | 3.90 (2.31–6.59) | 4.00 (2.34–6.83) | 4.34 (2.59–7.29) |
|  | Substance Use Disorder | 4.74 (3.79–5.92) | 4.94 (3.95–6.16) | 4.92 (3.94–6.14) | 4.84 (3.88–6.05) | 4.44 (3.54–5.58) | 4.10 (3.27–5.16) | 4.88 (3.91–6.08) |
|  | Personality Disorder | 1.64 (0.82–3.31) | 1.61 (0.80–3.27) | 1.62 (0.80–3.29) | 1.44 (0.72–2.91) | 1.35 (0.65–2.79) | 1.38 (0.67–2.83) | 1.68 (0.83–3.39) |

Complete fully adjusted model outputs for CNS medications separately, including estimates for all covariates one month before death by suicide among women.

***Supplementary Table S6: Adjusted Odds Ratios of Selected CNS Medication Use for Women in the Three Months Period Before Death by Suicide***

|  |  | Opioids | Minor analgesics and antipyretics | Antiepileptics | Antipsychotics | Anxiolytics | Hypnotics and sedatives | Antidepressants |
| --- | --- | --- | --- | --- | --- | --- | --- | --- |
|  |  | 1.88 (1.63–2.17) | 0.95 (0.84–1.09) | 1.11 (0.84–1.49) | 2.03 (1.66–2.49) | 3.41 (2.99–3.88) | 3.67 (3.27–4.13) | 2.27 (2.00–2.57) |
| Education | Primary | Ref | Ref | Ref | Ref | Ref | Ref | Ref |
|  | Secondary | 1.02 (0.90–1.16) | 1.02 (0.90–1.16) | 1.02 (0.90–1.16) | 1.03 (0.91–1.17) | 1.04 (0.92–1.18) | 0.99 (0.87–1.12) | 1.03 (0.91–1.17) |
|  | Tertiary | 1.16 (1.00–1.35) | 1.15 (0.99–1.34) | 1.16 (1.00–1.34) | 1.17 (1.01–1.36) | 1.21 (1.04–1.40) | 1.10 (0.95–1.28) | 1.16 (1.00–1.35) |
|  | Not available | 1.26 (0.89–1.77) | 1.29 (0.91–1.81) | 1.27 (0.91–1.79) | 1.28 (0.91–1.80) | 1.36 (0.96–1.92) | 1.35 (0.95–1.91) | 1.32 (0.93–1.85) |
| Civil Status | Married or Partnered | Ref | Ref | Ref | Ref | Ref | Ref | Ref |
|  | Single or Divorced | 2.32 (2.04–2.65) | 2.37 (2.08–2.71) | 2.36 (2.07–2.70) | 2.34 (2.05–2.67) | 2.26 (1.98–2.58) | 2.28 (2.00–2.61) | 2.34 (2.06–2.67) |
|  | Widow | 1.58 (1.36–1.84) | 1.60 (1.38–1.86) | 1.59 (1.37–1.85) | 1.59 (1.37–1.85) | 1.56 (1.34–1.81) | 1.54 (1.33–1.80) | 1.55 (1.34–1.81) |
|  | Data not available | 0.07 (0.01–0.59) | 0.08 (0.01–0.62) | 0.08 (0.01–0.62) | 0.08 (0.01–0.62) | 0.06 (0.01–0.55) | 0.07 (0.01–0.62) | 0.09 (0.01–0.69) |
| Frailty Risk Score | Low Frailty Risk | Ref | Ref | Ref | Ref | Ref | Ref | Ref |
|  | Medium Frailty Risk | 1.39 (1.18–1.64) | 1.44 (1.22–1.69) | 1.43 (1.22–1.68) | 1.44 (1.22–1.69) | 1.41 (1.20–1.67) | 1.47 (1.25–1.73) | 1.40 (1.19–1.65) |
|  | High Frailty Risk | 1.22 (1.00–1.49) | 1.31 (1.07–1.60) | 1.29 (1.06–1.58) | 1.27 (1.04–1.56) | 1.32 (1.08–1.62) | 1.44 (1.18–1.77) | 1.26 (1.04–1.54) |
| Number of Other Classes of Drugs |  | 1.04 (1.03–1.06) | 1.07 (1.06–1.09) | 1.07 (1.05–1.08) | 1.06 (1.05–1.07) | 1.03 (1.01–1.04) | 1.01 (1.00–1.03) | 1.04 (1.03–1.05) |
| Self-harm History | Yes | 11.78 (9.71–14.29) | 12.03 (9.93–14.58) | 12.02 (9.92–14.57) | 12.06 (9.95–14.62) | 11.56 (9.50–14.07) | 11.01 (9.04–13.41) | 11.34 (9.35–13.76) |
| Psychaitric diagnosis | Depression | 6.42 (5.46–7.55) | 6.26 (5.32–7.35) | 6.25 (5.32–7.35) | 5.86 (4.98–6.89) | 5.50 (4.67–6.48) | 5.22 (4.43–6.16) | 4.64 (3.94–5.48) |
|  | Anxiety | 3.49 (2.88–4.21) | 3.44 (2.85–4.16) | 3.44 (2.85–4.16) | 3.28 (2.71–3.96) | 2.31 (1.90–2.81) | 2.85 (2.35–3.45) | 2.88 (2.39–3.48) |
|  | Bipolar Disorder | 3.36 (2.37–4.77) | 3.19 (2.25–4.53) | 3.18 (2.22–4.55) | 2.23 (1.54–3.21) | 2.75 (1.92–3.94) | 2.57 (1.79–3.68) | 2.79 (1.96–3.96) |
|  | Schizophrenia | 4.50 (2.70–7.51) | 4.39 (2.63–7.31) | 4.35 (2.61–7.24) | 2.70 (1.58–4.60) | 3.93 (2.33–6.61) | 4.07 (2.39–6.92) | 4.29 (2.55–7.23) |
|  | Substance Use Disorder | 4.65 (3.72–5.82) | 4.86 (3.90–6.07) | 4.86 (3.89–6.07) | 4.78 (3.83–5.97) | 4.48 (3.57–5.62) | 4.13 (3.29–5.18) | 4.78 (3.84–5.96) |
|  | Personality Disorder | 1.62 (0.81–3.26) | 1.57 (0.78–3.18) | 1.58 (0.78–3.19) | 1.39 (0.69–2.78) | 1.32 (0.64–2.73) | 1.40 (0.68–2.87) | 1.64 (0.82–3.28) |

Complete fully adjusted model outputs for CNS medications separately, including estimates for all covariates three months before death by suicide among women.

***Supplementary Table S7: Adjusted Odds Ratios of Selected CNS Medication Use for Women in the One Year Period Before Death by Suicide***

|  |  | Opioids | Minor analgesics and antipyretics | Antiepileptics | Antipsychotics | Anxiolytics | Hypnotics and sedatives | Antidepressants |
| --- | --- | --- | --- | --- | --- | --- | --- | --- |
|  |  | 1.78 (1.55–2.04) | 0.97 (0.85–1.10) | 1.10 (0.83–1.45) | 2.05 (1.68–2.50) | 3.28 (2.88–3.72) | 3.68 (3.27–4.14) | 2.35 (2.07–2.66) |
| Education | Primary | Ref | Ref | Ref | Ref | Ref | Ref | Ref |
|  | Secondary | 1.02 (0.90–1.16) | 1.02 (0.90–1.16) | 1.02 (0.90–1.16) | 1.03 (0.90–1.16) | 1.04 (0.92–1.18) | 0.99 (0.87–1.12) | 1.03 (0.91–1.17) |
|  | Tertiary | 1.15 (1.00–1.34) | 1.14 (0.99–1.33) | 1.15 (0.99–1.33) | 1.16 (1.00–1.35) | 1.20 (1.03–1.39) | 1.09 (0.94–1.26) | 1.16 (1.00–1.35) |
|  | Not available | 1.25 (0.89–1.76) | 1.28 (0.91–1.80) | 1.27 (0.90–1.79) | 1.27 (0.91–1.79) | 1.33 (0.94–1.87) | 1.35 (0.95–1.92) | 1.31 (0.93–1.85) |
| Civil Status | Married or Partnered | Ref | Ref | Ref | Ref | Ref | Ref | Ref |
|  | Single or Divorced | 2.33 (2.04–2.65) | 2.37 (2.07–2.70) | 2.36 (2.07–2.69) | 2.34 (2.05–2.67) | 2.25 (1.97–2.57) | 2.28 (2.00–2.61) | 2.33 (2.05–2.66) |
|  | Widow | 1.58 (1.36–1.84) | 1.60 (1.38–1.86) | 1.59 (1.37–1.85) | 1.59 (1.37–1.85) | 1.55 (1.33–1.80) | 1.54 (1.33–1.80) | 1.55 (1.34–1.81) |
|  | Data not available | 0.07 (0.01–0.59) | 0.07 (0.01–0.61) | 0.07 (0.01–0.61) | 0.07 (0.01–0.62) | 0.06 (0.01–0.56) | 0.07 (0.01–0.64) | 0.09 (0.01–0.70) |
| Frailty Risk Score | Low Frailty Risk | Ref | Ref | Ref | Ref | Ref | Ref | Ref |
|  | Medium Frailty Risk | 1.38 (1.18–1.63) | 1.43 (1.22–1.68) | 1.42 (1.21–1.67) | 1.43 (1.21–1.68) | 1.41 (1.20–1.66) | 1.46 (1.24–1.72) | 1.39 (1.18–1.64) |
|  | High Frailty Risk | 1.21 (0.99–1.48) | 1.29 (1.06–1.58) | 1.28 (1.04–1.56) | 1.26 (1.03–1.54) | 1.30 (1.06–1.59) | 1.42 (1.16–1.73) | 1.25 (1.02–1.52) |
| Number of Other Classes of Drugs |  | 1.04 (1.03–1.06) | 1.07 (1.05–1.08) | 1.06 (1.05–1.07) | 1.06 (1.05–1.07) | 1.03 (1.01–1.04) | 1.01 (1.00–1.03) | 1.04 (1.02–1.05) |
| Self-harm History | Yes | 11.84 (9.76–14.36) | 12.05 (9.95–14.61) | 12.04 (9.94–14.60) | 12.09 (9.97–14.65) | 11.53 (9.47–14.02) | 10.96 (9.00–13.34) | 11.28 (9.30–13.68) |
| Psychaitric diagnosis | Depression | 6.38 (5.43–7.50) | 6.23 (5.30–7.33) | 6.23 (5.30–7.33) | 5.78 (4.91–6.80) | 5.38 (4.57–6.33) | 5.20 (4.41–6.13) | 4.56 (3.86–5.38) |
|  | Anxiety | 3.48 (2.88–4.20) | 3.44 (2.85–4.16) | 3.44 (2.85–4.16) | 3.26 (2.69–3.94) | 2.34 (1.93–2.84) | 2.86 (2.36–3.46) | 2.86 (2.37–3.45) |
|  | Bipolar Disorder | 3.36 (2.36–4.76) | 3.20 (2.26–4.54) | 3.19 (2.23–4.57) | 2.20 (1.53–3.18) | 2.83 (1.98–4.04) | 2.55 (1.78–3.65) | 2.77 (1.95–3.94) |
|  | Schizophrenia | 4.56 (2.74–7.61) | 4.42 (2.65–7.37) | 4.38 (2.63–7.31) | 2.69 (1.58–4.59) | 3.89 (2.32–6.53) | 4.12 (2.42–7.01) | 4.35 (2.58–7.33) |
|  | Substance Use Disorder | 4.66 (3.73–5.83) | 4.82 (3.86–6.01) | 4.81 (3.85–6.00) | 4.74 (3.80–5.93) | 4.50 (3.59–5.65) | 4.08 (3.25–5.13) | 4.70 (3.77–5.86) |
|  | Personality Disorder | 1.62 (0.80–3.26) | 1.59 (0.79–3.23) | 1.59 (0.78–3.22) | 1.32 (0.66–2.66) | 1.22 (0.60–2.50) | 1.43 (0.70–2.94) | 1.66 (0.83–3.32) |

Complete fully adjusted model outputs for CNS medications separately, including estimates for all covariates one year before death by suicide among women.

***Supplementary Table S8: Adjusted Odds Ratios of Selected CNS Medication Use for Men in the One Month Period Before Death by Suicide***

|  |  | Opioids | Minor analgesics and antipyretics | Antiepileptics | Antipsychotics | Anxiolytics | Hypnotics and sedatives | Antidepressants |
| --- | --- | --- | --- | --- | --- | --- | --- | --- |
|  |  | 2.34 (2.12–2.59) | 1.16 (1.05–1.27) | 1.21 (1.00–1.46) | 1.40 (1.18–1.64) | 3.63 (3.30–3.98) | 3.66 (3.38–3.96) | 2.49 (2.28–2.72) |
| Education | Primary | Ref | Ref | Ref | Ref | Ref | Ref | Ref |
|  | Secondary | 0.87 (0.81–0.94) | 0.87 (0.80–0.93) | 0.87 (0.80–0.93) | 0.87 (0.80–0.93) | 0.87 (0.81–0.94) | 0.85 (0.78–0.91) | 0.86 (0.80–0.93) |
|  | Tertiary | 0.74 (0.68–0.82) | 0.74 (0.67–0.82) | 0.74 (0.67–0.81) | 0.74 (0.67–0.81) | 0.74 (0.67–0.81) | 0.70 (0.64–0.77) | 0.74 (0.67–0.81) |
|  | Not available | 0.77 (0.60–0.98) | 0.78 (0.61–1.00) | 0.78 (0.61–1.00) | 0.78 (0.61–0.99) | 0.76 (0.59–0.97) | 0.76 (0.59–0.97) | 0.79 (0.62–1.01) |
| Civil Status | Married or Partnered | Ref | Ref | Ref | Ref | Ref | Ref | Ref |
|  | Single or Divorced | 2.36 (2.19–2.54) | 2.39 (2.22–2.58) | 2.40 (2.23–2.58) | 2.39 (2.22–2.57) | 2.32 (2.15–2.50) | 2.30 (2.14–2.48) | 2.37 (2.20–2.55) |
|  | Widow | 2.00 (1.80–2.21) | 2.01 (1.81–2.22) | 2.01 (1.81–2.23) | 2.01 (1.82–2.23) | 2.00 (1.80–2.21) | 1.96 (1.77–2.17) | 1.97 (1.78–2.19) |
|  | Data not available | 0.24 (0.12–0.47) | 0.25 (0.13–0.49) | 0.25 (0.13–0.49) | 0.25 (0.13–0.49) | 0.24 (0.12–0.47) | 0.24 (0.12–0.48) | 0.24 (0.12–0.48) |
| Frailty Risk Score | Low Frailty Risk | Ref | Ref | Ref | Ref | Ref | Ref | Ref |
|  | Medium Frailty Risk | 1.32 (1.18–1.46) | 1.34 (1.20–1.48) | 1.34 (1.21–1.49) | 1.34 (1.21–1.49) | 1.34 (1.21–1.49) | 1.33 (1.20–1.48) | 1.28 (1.15–1.42) |
|  | High Frailty Risk | 1.00 (0.87–1.14) | 1.05 (0.91–1.20) | 1.05 (0.92–1.20) | 1.05 (0.92–1.20) | 1.04 (0.91–1.19) | 1.03 (0.90–1.18) | 0.98 (0.86–1.12) |
| Number of Other Classes of Drugs |  | 1.04 (1.03–1.05) | 1.06 (1.05–1.07) | 1.06 (1.05–1.07) | 1.06 (1.05–1.07) | 1.03 (1.02–1.04) | 1.01 (1.00–1.02) | 1.03 (1.02–1.04) |
| Self-harm History | Yes | 5.35 (4.60–6.21) | 5.38 (4.63–6.24) | 5.38 (4.64–6.25) | 5.37 (4.63–6.23) | 5.32 (4.58–6.19) | 4.93 (4.23–5.75) | 5.22 (4.50–6.06) |
| Psychaitric diagnosis | Depression | 6.54 (5.79–7.38) | 6.42 (5.70–7.24) | 6.41 (5.69–7.23) | 6.24 (5.53–7.04) | 5.47 (4.84–6.18) | 5.32 (4.71–6.01) | 4.32 (3.81–4.90) |
|  | Anxiety | 2.77 (2.34–3.27) | 2.73 (2.31–3.23) | 2.73 (2.31–3.23) | 2.69 (2.28–3.18) | 1.92 (1.61–2.27) | 2.29 (1.94–2.72) | 2.28 (1.93–2.70) |
|  | Bipolar Disorder | 3.23 (2.45–4.26) | 3.08 (2.34–4.06) | 2.96 (2.24–3.92) | 2.66 (2.00–3.53) | 2.70 (2.05–3.55) | 2.51 (1.90–3.32) | 2.66 (2.02–3.50) |
|  | Schizophrenia | 1.80 (1.17–2.75) | 1.69 (1.11–2.58) | 1.68 (1.10–2.56) | 1.38 (0.89–2.14) | 1.33 (0.87–2.05) | 1.39 (0.91–2.13) | 1.55 (1.02–2.37) |
|  | Substance Use Disorder | 3.15 (2.78–3.58) | 3.21 (2.83–3.63) | 3.21 (2.83–3.63) | 3.22 (2.85–3.65) | 3.10 (2.73–3.51) | 2.88 (2.54–3.27) | 3.21 (2.84–3.64) |
|  | Personality Disorder | 1.65 (0.85–3.20) | 1.61 (0.83–3.11) | 1.61 (0.83–3.12) | 1.52 (0.79–2.93) | 1.38 (0.71–2.69) | 1.22 (0.62–2.39) | 1.57 (0.82–3.01) |

Complete fully adjusted model outputs for CNS medications separately, including estimates for all covariates one month before death by suicide among men.

***Supplementary Table S9: Adjusted Odds Ratios of Selected CNS Medication Use for Men in the Three Months Period Before Death by Suicide***

|  |  | Opioids | Minor analgesics and antipyretics | Antiepileptics | Antipsychotics | Anxiolytics | Hypnotics and sedatives | Antidepressants |
| --- | --- | --- | --- | --- | --- | --- | --- | --- |
|  |  | 2.15 (1.96–2.37) | 1.14 (1.05–1.25) | 1.22 (1.02–1.47) | 1.57 (1.35–1.84) | 3.47 (3.17–3.80) | 3.60 (3.33–3.90) | 2.56 (2.35–2.80) |
| Education | Primary | Ref | Ref | Ref | Ref | Ref | Ref | Ref |
|  | Secondary | 0.87 (0.81–0.94) | 0.87 (0.80–0.93) | 0.86 (0.80–0.93) | 0.87 (0.80–0.93) | 0.87 (0.81–0.94) | 0.84 (0.78–0.91) | 0.86 (0.80–0.93) |
|  | Tertiary | 0.74 (0.68–0.82) | 0.74 (0.67–0.81) | 0.74 (0.67–0.81) | 0.74 (0.67–0.81) | 0.74 (0.67–0.81) | 0.70 (0.64–0.77) | 0.74 (0.67–0.81) |
|  | Not available | 0.77 (0.60–0.98) | 0.78 (0.61–0.99) | 0.78 (0.61–0.99) | 0.77 (0.61–0.99) | 0.76 (0.60–0.97) | 0.75 (0.59–0.96) | 0.79 (0.62–1.01) |
| Civil Status | Married or Partnered | Ref | Ref | Ref | Ref | Ref | Ref | Ref |
|  | Single or Divorced | 2.37 (2.20–2.55) | 2.40 (2.23–2.58) | 2.40 (2.23–2.58) | 2.38 (2.21–2.57) | 2.32 (2.16–2.50) | 2.30 (2.13–2.48) | 2.37 (2.20–2.55) |
|  | Widow | 2.00 (1.80–2.21) | 2.01 (1.81–2.22) | 2.01 (1.81–2.22) | 2.01 (1.82–2.23) | 1.99 (1.79–2.20) | 1.96 (1.77–2.17) | 1.97 (1.78–2.18) |
|  | Data not available | 0.25 (0.13–0.49) | 0.26 (0.13–0.50) | 0.26 (0.13–0.50) | 0.26 (0.13–0.50) | 0.24 (0.12–0.48) | 0.25 (0.13–0.49) | 0.25 (0.13–0.49) |
| Frailty Risk Score | Low Frailty Risk | Ref | Ref | Ref | Ref | Ref | Ref | Ref |
|  | Medium Frailty Risk | 1.29 (1.16–1.44) | 1.31 (1.18–1.46) | 1.31 (1.18–1.46) | 1.32 (1.19–1.46) | 1.31 (1.18–1.46) | 1.31 (1.18–1.46) | 1.25 (1.12–1.39) |
|  | High Frailty Risk | 0.98 (0.86–1.12) | 1.02 (0.90–1.17) | 1.02 (0.90–1.17) | 1.02 (0.89–1.17) | 1.02 (0.89–1.17) | 1.01 (0.88–1.15) | 0.96 (0.84–1.09) |
| Number of Other Classes of Drugs |  | 1.04 (1.03–1.05) | 1.06 (1.05–1.07) | 1.06 (1.05–1.07) | 1.06 (1.05–1.07) | 1.03 (1.02–1.04) | 1.01 (1.00–1.02) | 1.04 (1.03–1.04) |
| Self-harm History | Yes | 5.33 (4.59–6.18) | 5.37 (4.63–6.23) | 5.37 (4.63–6.23) | 5.36 (4.62–6.21) | 5.27 (4.53–6.13) | 4.92 (4.23–5.74) | 5.20 (4.48–6.04) |
| Psychaitric diagnosis | Depression | 6.46 (5.73–7.29) | 6.39 (5.66–7.20) | 6.37 (5.65–7.19) | 6.11 (5.42–6.90) | 5.34 (4.72–6.03) | 5.26 (4.66–5.95) | 4.21 (3.72–4.78) |
|  | Anxiety | 2.74 (2.32–3.24) | 2.70 (2.29–3.19) | 2.70 (2.29–3.19) | 2.64 (2.23–3.11) | 1.90 (1.61–2.26) | 2.25 (1.90–2.67) | 2.22 (1.88–2.62) |
|  | Bipolar Disorder | 3.19 (2.42–4.21) | 3.05 (2.31–4.01) | 2.92 (2.21–3.86) | 2.44 (1.83–3.24) | 2.66 (2.02–3.50) | 2.46 (1.86–3.25) | 2.61 (1.98–3.44) |
|  | Schizophrenia | 1.78 (1.17–2.73) | 1.67 (1.10–2.55) | 1.66 (1.09–2.54) | 1.25 (0.81–1.94) | 1.33 (0.87–2.05) | 1.37 (0.90–2.09) | 1.54 (1.01–2.35) |
|  | Substance Use Disorder | 3.14 (2.77–3.55) | 3.19 (2.82–3.61) | 3.19 (2.82–3.61) | 3.21 (2.83–3.63) | 3.09 (2.72–3.50) | 2.85 (2.51–3.23) | 3.17 (2.80–3.59) |
|  | Personality Disorder | 1.68 (0.86–3.27) | 1.60 (0.83–3.10) | 1.61 (0.83–3.11) | 1.45 (0.75–2.78) | 1.37 (0.70–2.68) | 1.24 (0.63–2.43) | 1.51 (0.79–2.90) |

Complete fully adjusted model outputs for CNS medications separately, including estimates for all covariates three months before death by suicide among men.

***Supplementary Table S10: Adjusted Odds Ratios of Selected CNS Medication Use for Men in the One Year Period Before Death by Suicide***

|  |  | Opioids | Minor analgesics and antipyretics | Antiepileptics | Antipsychotics | Anxiolytics | Hypnotics and sedatives | Antidepressants |
| --- | --- | --- | --- | --- | --- | --- | --- | --- |
|  |  | 2.00 (1.82–2.19) | 1.12 (1.03–1.22) | 1.27 (1.07–1.52) | 1.63 (1.40–1.89) | 3.29 (3.01–3.60) | 3.52 (3.25–3.80) | 2.61 (2.40–2.85) |
| Education | Primary | Ref | Ref | Ref | Ref | Ref | Ref | Ref |
|  | Secondary | 0.87 (0.80–0.93) | 0.86 (0.80–0.93) | 0.86 (0.80–0.93) | 0.86 (0.80–0.93) | 0.87 (0.81–0.94) | 0.84 (0.78–0.91) | 0.86 (0.80–0.93) |
|  | Tertiary | 0.74 (0.67–0.81) | 0.74 (0.67–0.81) | 0.73 (0.67–0.81) | 0.74 (0.67–0.81) | 0.74 (0.67–0.81) | 0.70 (0.64–0.77) | 0.73 (0.67–0.81) |
|  | Not available | 0.76 (0.60–0.97) | 0.77 (0.61–0.99) | 0.77 (0.61–0.99) | 0.77 (0.60–0.98) | 0.76 (0.60–0.98) | 0.75 (0.59–0.96) | 0.79 (0.62–1.01) |
| Civil Status | Married or Partnered | Ref | Ref | Ref | Ref | Ref | Ref | Ref |
|  | Single or Divorced | 2.38 (2.21–2.56) | 2.40 (2.23–2.59) | 2.40 (2.23–2.58) | 2.39 (2.22–2.57) | 2.33 (2.16–2.51) | 2.30 (2.13–2.48) | 2.37 (2.20–2.55) |
|  | Widow | 2.00 (1.81–2.22) | 2.01 (1.81–2.23) | 2.01 (1.81–2.23) | 2.01 (1.82–2.23) | 1.98 (1.79–2.19) | 1.95 (1.76–2.16) | 1.97 (1.78–2.18) |
|  | Data not available | 0.25 (0.13–0.50) | 0.26 (0.13–0.51) | 0.26 (0.13–0.51) | 0.26 (0.13–0.51) | 0.25 (0.13–0.48) | 0.25 (0.13–0.49) | 0.25 (0.13–0.50) |
| Frailty Risk Score | Low Frailty Risk | Ref | Ref | Ref | Ref | Ref | Ref | Ref |
|  | Medium Frailty Risk | 1.28 (1.15–1.42) | 1.30 (1.17–1.45) | 1.30 (1.17–1.44) | 1.30 (1.17–1.45) | 1.30 (1.17–1.44) | 1.31 (1.18–1.45) | 1.24 (1.11–1.37) |
|  | High Frailty Risk | 0.97 (0.85–1.11) | 1.01 (0.89–1.16) | 1.01 (0.88–1.16) | 1.00 (0.88–1.15) | 1.01 (0.88–1.15) | 1.00 (0.88–1.15) | 0.94 (0.82–1.08) |
| Number of Other Classes of Drugs |  | 1.04 (1.03–1.04) | 1.06 (1.05–1.06) | 1.06 (1.05–1.07) | 1.06 (1.05–1.06) | 1.03 (1.02–1.04) | 1.01 (1.00–1.02) | 1.03 (1.02–1.04) |
| Self-harm History | Yes | 5.34 (4.60–6.19) | 5.37 (4.63–6.23) | 5.37 (4.63–6.23) | 5.36 (4.62–6.22) | 5.26 (4.52–6.12) | 4.91 (4.22–5.72) | 5.20 (4.48–6.04) |
| Psychaitric diagnosis | Depression | 6.49 (5.75–7.32) | 6.39 (5.67–7.21) | 6.38 (5.66–7.19) | 6.07 (5.38–6.86) | 5.35 (4.74–6.04) | 5.25 (4.65–5.93) | 4.15 (3.66–4.70) |
|  | Anxiety | 2.73 (2.31–3.22) | 2.69 (2.28–3.17) | 2.68 (2.27–3.17) | 2.61 (2.21–3.08) | 1.90 (1.60–2.25) | 2.24 (1.89–2.65) | 2.19 (1.86–2.59) |
|  | Bipolar Disorder | 3.17 (2.41–4.17) | 3.04 (2.31–4.00) | 2.88 (2.18–3.81) | 2.38 (1.79–3.16) | 2.63 (2.00–3.47) | 2.48 (1.88–3.27) | 2.59 (1.97–3.42) |
|  | Schizophrenia | 1.80 (1.18–2.76) | 1.69 (1.11–2.58) | 1.68 (1.10–2.56) | 1.22 (0.79–1.89) | 1.36 (0.89–2.09) | 1.37 (0.90–2.09) | 1.55 (1.02–2.37) |
|  | Substance Use Disorder | 3.13 (2.76–3.55) | 3.18 (2.81–3.60) | 3.18 (2.81–3.60) | 3.20 (2.83–3.62) | 3.08 (2.71–3.49) | 2.87 (2.53–3.26) | 3.14 (2.78–3.56) |
|  | Personality Disorder | 1.65 (0.85–3.20) | 1.59 (0.82–3.09) | 1.60 (0.83–3.09) | 1.43 (0.75–2.74) | 1.41 (0.73–2.75) | 1.29 (0.66–2.51) | 1.49 (0.78–2.85) |

Complete fully adjusted model outputs for CNS medications separately, including estimates for all covariates one year before death by suicide among men.

***Supplementary Table S11: Descriptive Table of Selected CNS Drugs***

|  | One Month Before Suicide | | | | Three Months Before Suicide | | | | One Year Before Suicide | | | |
| --- | --- | --- | --- | --- | --- | --- | --- | --- | --- | --- | --- | --- |
|  | Case | | Control | | Case | | Control | | Case | | Control | |
| Drug | N | % | N | % | N | % | N | % | N | % | N | % |
| Hypnotics and sedatives (N05C) | 2580 | 43.2 | 20770 | 11.6 | 2691 | 45.1 | 22545 | 12.6 | 2776 | 46.5 | 24234 | 13.5 |
| Antidepressants (N06A) | 2128 | 35.6 | 17047 | 9.5 | 2238 | 37.5 | 17950 | 10.0 | 2316 | 38.8 | 18714 | 10.4 |
| Anxiolytics (N05B) | 1700 | 28.5 | 9070 | 5.1 | 1827 | 30.6 | 10530 | 5.9 | 1929 | 32.3 | 12094 | 6.8 |
| Minor analgesics and antipyretics (N02B) | 1376 | 23.0 | 25163 | 14.0 | 1589 | 26.6 | 29948 | 16.7 | 1787 | 29.9 | 34510 | 19.3 |
| Opioids (N02A) | 1076 | 18.0 | 10601 | 5.9 | 1227 | 20.5 | 13246 | 7.4 | 1391 | 23.3 | 16674 | 9.3 |
| Antipsychotics (N05A) | 557 | 9.3 | 3241 | 1.8 | 618 | 10.4 | 3428 | 1.9 | 662 | 11.1 | 3599 | 2.0 |
| Antiepileptics (N03A) | 255 | 4.3 | 2778 | 1.6 | 278 | 4.7 | 2938 | 1.6 | 302 | 5.1 | 3086 | 1.7 |

The prevalence of CNS drugs used in the study population during different time windows.

***Supplementary Table S12: Descriptive Table of Selected CNS Drugs in Women***

| Women | One Month Before Suicide | | | | Three Months Before Suicide | | | | One Year Before Suicide | | | |
| --- | --- | --- | --- | --- | --- | --- | --- | --- | --- | --- | --- | --- |
|  | Case | | Control | | Case | | Control | | Case | | Control | |
| Drug | N | % | N | % | N | % | N | % | N | % | N | % |
| Hypnotics and sedatives (N05C) | 1016 | 55 | 8684 | 15.7 | 1051 | 56.9 | 9418 | 17 | 1087 | 58.9 | 10114 | 18.3 |
| Antidepressants (N06A) | 852 | 46.2 | 7440 | 13.4 | 894 | 48.4 | 7831 | 14.1 | 926 | 50.2 | 8151 | 14.7 |
| Anxiolytics (N05B) | 715 | 38.7 | 3879 | 7 | 767 | 41.5 | 4579 | 8.3 | 808 | 43.8 | 5252 | 9.5 |
| Minor analgesics and antipyretics (N02B) | 529 | 28.7 | 10221 | 18.5 | 610 | 33 | 12043 | 21.7 | 683 | 37 | 13631 | 24.6 |
| Opioids (N02A) | 405 | 21.9 | 4103 | 7.4 | 456 | 24.7 | 5005 | 9 | 511 | 27.7 | 6157 | 11.1 |
| Antipsychotics (N05A) | 260 | 14.1 | 1230 | 2.2 | 280 | 15.2 | 1292 | 2.3 | 299 | 16.2 | 1348 | 2.4 |
| Antiepileptics (N03A) | 90 | 4.9 | 761 | 1.4 | 102 | 5.5 | 806 | 1.5 | 112 | 6.1 | 864 | 1.6 |

The prevalence of CNS drugs used in women during different time windows.

***Supplementary Table S13: Descriptive Table of Selected CNS Drugs in Men***

| \|  \| One Month Before Suicide \| \| \| \| Three Months Before Suicide \| \| \| \| One Year Before Suicide \| \| \| \| \| --- \| --- \| --- \| --- \| --- \| --- \| --- \| --- \| --- \| --- \| --- \| --- \| --- \| \|  \| Case \| \| Control \| \| Case \| \| Control \| \| Case \| \| Control \| \| \| Drug \| N \| % \| N \| % \| N \| % \| N \| % \| N \| % \| N \| % \| \| Hypnotics and sedatives (N05C) \| 1564 \| 37.9 \| 12086 \| 9.8 \| 1640 \| 39.8 \| 13127 \| 10.6 \| 1689 \| 40.9 \| 14120 \| 11.4 \| \| Antidepressants (N06A) \| 1276 \| 30.9 \| 9607 \| 7.8 \| 1344 \| 32.6 \| 10119 \| 8.2 \| 1390 \| 33.7 \| 10563 \| 8.5 \| \| Anxiolytics (N05B) \| 985 \| 23.9 \| 5191 \| 4.2 \| 1060 \| 25.7 \| 5951 \| 4.8 \| 1121 \| 27.2 \| 6842 \| 5.5 \| \| Minor analgesics and antipyretics (N02B) \| 847 \| 20.5 \| 14942 \| 12.1 \| 979 \| 23.7 \| 17905 \| 14.5 \| 1104 \| 26.8 \| 20879 \| 16.9 \| \| Opioids (N02A) \| 671 \| 16.3 \| 6498 \| 5.3 \| 771 \| 18.7 \| 8241 \| 6.7 \| 880 \| 21.3 \| 10517 \| 8.5 \| \| Antipsychotics (N05A) \| 297 \| 7.2 \| 2011 \| 1.6 \| 338 \| 8.2 \| 2136 \| 1.7 \| 363 \| 8.8 \| 2251 \| 1.8 \| \| Antiepileptics (N03A) \| 165 \| 4 \| 2017 \| 1.6 \| 176 \| 4.3 \| 2132 \| 1.7 \| 190 \| 4.6 \| 2222 \| 1.8 \| |  |  |  |
| --- | --- | --- | --- | --- | --- | --- | --- | --- | --- | --- | --- | --- | --- | --- | --- | --- | --- | --- | --- | --- | --- | --- | --- | --- | --- | --- | --- | --- | --- | --- | --- | --- | --- | --- | --- | --- | --- | --- | --- | --- | --- | --- | --- | --- | --- | --- | --- | --- | --- | --- | --- | --- | --- | --- | --- | --- | --- | --- | --- | --- | --- | --- | --- | --- | --- | --- | --- | --- | --- | --- | --- | --- | --- | --- | --- | --- | --- | --- | --- | --- | --- | --- | --- | --- | --- | --- | --- | --- | --- | --- | --- | --- | --- | --- | --- | --- | --- | --- | --- | --- | --- | --- | --- | --- | --- | --- | --- | --- | --- | --- | --- | --- | --- | --- | --- | --- | --- | --- | --- | --- | --- | --- | --- | --- | --- | --- | --- | --- | --- | --- | --- | --- | --- |

The prevalence of CNS drugs used in men during different time windows.

***Supplementary Table S14: Sensitivity Analysis***

| **Drug** | **Main analysis (%)^a^** | **Sensitivity analysis (%)^b^** | **Main analysis (%)^a^** | **Sensitivity analysis (%)^b^** |
| --- | --- | --- | --- | --- |
| Hypnotics and sedatives | 46.5 | 46.7 | 13.5 | 17.7 |
| Antidepressants | 38.8 | 39 | 10.4 | 13.3 |
| Anxiolytics | 32.3 | 32.4 | 6.8 | 9.6 |
| Other analgesics and antipyretics | 29.9 | 30 | 19.3 | 27 |
| Opioids | 23.3 | 23.2 | 9.3 | 14.2 |
| Antipsychotics | 11.1 | 11 | 2 | 2.6 |
| Antiepileptics | 5.1 | 5 | 1.7 | 2.1 |

1. Prevalance calculated according to our algorithm.^7^
2. Prevalence calculated according to traditional ddd methods.

In the sensitivity analysis, drug exposure duration was defined based on defined daily dose (DDD) rather than the algorithm-estimated duration. We calculated the total amount of DDDs dispensed and divided this by the standard DDD to derive the duration of medication exposure.

***Supplementary Table S15: RECORD Statement***

The RECORD statement for pharmacoepidemiology (RECORD-PE) checklist of items, extended from the STROBE and RECORD statements, which should be reported in non-interventional pharmacoepidemiological studies using routinely collected health data

| Item No | STROBE items | RECORD items | RECORD-PE items | Page No |
| --- | --- | --- | --- | --- |
| **Title and abstract** | | | | |
| 1 | (a) Indicate the study’s design with a commonly used term in the title or the abstract.  (b) Provide in the abstract an informative and balanced summary of what was done and what was found. | 1.1: The type of data used should be specified in the title or abstract. When possible, the name of the databases used should be included.  1.2: If applicable, the geographical region and timeframe within which the study took place should be reported in the title or abstract.  1.3: If linkage between databases was conducted for the study, this should be clearly stated in the title or abstract. | — | 1-3 |
| **Introduction** | | | | |
| Background rationale | | | | |
| 2 | Explain the scientific background and rationale for the investigation being reported. | — | — | 4 |
| Objectives | | | | |
| 3 | State specific objectives, including any prespecified hypotheses. | — | — | 4 |
| **Methods** | | | | |
| Study design | | | | |
| 4 | Present key elements of study design early in the paper. | — | 4.a: Include details of the specific study design (and its features) and report the use of multiple designs if used.  4.b: The use of a diagram(s) is recommended to illustrate key aspects of the study design(s), including exposure, washout, lag and observation periods, and covariate definitions as relevant. | 6 |
| Setting | | | | |
| 5 | Describe the setting, locations, and relevant dates, including periods of recruitment, exposure, follow-up, and data collection. | — | — | 5 |
| Participants | | | | |
| 6 | (a) Cohort study—give the eligibility criteria, and the sources and methods of selection of participants. Describe methods of follow-up. Case-control study—give the eligibility criteria, and the sources and methods of case ascertainment and control selection. Give the rationale for the choice of cases and controls. Cross sectional study—give the eligibility criteria, and the sources and methods of selection of participants.  (b) Cohort study—for matched studies, give matching criteria and number of exposed and unexposed. Case-control study—for matched studies, give matching criteria and the number of controls per case. | 6.1: The methods of study population selection (such as codes or algorithms used to identify participants) should be listed in detail. If this is not possible, an explanation should be provided.  6.2: Any validation studies of the codes or algorithms used to select the population should be referenced. If validation was conducted for this study and not published elsewhere, detailed methods and results should be provided.  6.3: If the study involved linkage of databases, consider use of a flow diagram or other graphical display to demonstrate the data linkage process, including the number of individuals with linked data at each stage. | 6.1.a: Describe the study entry criteria and the order in which these criteria were applied to identify the study population. Specify whether only users with a specific indication were included and whether patients were allowed to enter the study population once or if multiple entries were permitted. See explanatory document for guidance related to matched designs. | 5 |
| Variables | | | | |
| 7 | Clearly define all outcomes, exposures, predictors, potential confounders, and effect modifiers. Give diagnostic criteria, if applicable. | 7.1: A complete list of codes and algorithms used to classify exposures, outcomes, confounders, and effect modifiers should be provided. If these cannot be reported, an explanation should be provided. | 7.1.a: Describe how the drug exposure definition was developed.  7.1.b: Specify the data sources from which drug exposure information for individuals was obtained.  7.1.c: Describe the time window(s) during which an individual is considered exposed to the drug(s). The rationale for selecting a particular time window should be provided. The extent of potential left truncation or left censoring should be specified.  7.1.d: Justify how events are attributed to current, prior, ever, or cumulative drug exposure.  7.1.e: When examining drug dose and risk attribution, describe how current, historical or time on therapy are considered.  7.1.f: Use of any comparator groups should be outlined and justified.  7.1.g: Outline the approach used to handle individuals with more than one relevant drug exposure during the study period. | 7-9 |
| Data sources/measurement | | | | |
| 8 | For each variable of interest, give sources of data and details of methods of assessment (measurement). Describe comparability of assessment methods if there is more than one group. | — | 8.a: Describe the healthcare system and mechanisms for generating the drug exposure records. Specify the care setting in which the drug(s) of interest was prescribed. | 5, 7 |
| Bias | | | | |
| 9 | Describe any efforts to address potential sources of bias. | — | — | 8-10 |
| Study size | | | | |
| 10 | Explain how the study size was arrived at. | — | — | 6 |
| Quantitative variables | | | | |
| 11 | Explain how quantitative variables were handled in the analyses. If applicable, describe which groupings were chosen, and why. | — | — | Not applicable |
| Statistical methods | | | | |
| 12 | (a) Describe all statistical methods, including those used to control for confounding.  (b) Describe any methods used to examine subgroups and interactions.  (c) Explain how missing data were addressed.  (d) Cohort study—if applicable, explain how loss to follow-up was addressed. Case-control study—if applicable, explain how matching of cases and controls was addressed. Cross sectional study—if applicable, describe analytical methods taking account of sampling strategy.  (e) Describe any sensitivity analyses. | — | 12.1.a: Describe the methods used to evaluate whether the assumptions have been met.  12.1.b: Describe and justify the use of multiple designs, design features, or analytical approaches. | 9-10 |
| Data access and cleaning methods | | | | |
| 12 | — | 12.1: Authors should describe the extent to which the investigators had access to the database population used to create the study population.  12.2: Authors should provide information on the data cleaning methods used in the study. | — | 5 |
| Linkage | | | | |
| 12 | — | 12.3: State whether the study included person level, institutional level, or other data linkage across two or more databases. The methods of linkage and methods of linkage quality evaluation should be provided. | — | 5 |
| **Results** | | | | |
| Participants | | | | |
| 13 | (a) Report the numbers of individuals at each stage of the study (eg, numbers potentially eligible, examined for eligibility, confirmed eligible, included in the study, completing follow-up, and analysed).  (b) Give reasons for non-participation at each stage.  (c) Consider use of a flow diagram. | 13.1: Describe in detail the selection of the individuals included in the study (that is, study population selection) including filtering based on data quality, data availability, and linkage. The selection of included individuals can be described in the text or by means of the study flow diagram. | — | 6 |
| Descriptive data | | | | |
| 14 | (a) Give characteristics of study participants (eg, demographic, clinical, social) and information on exposures and potential confounders.  (b) Indicate the number of participants with missing data for each variable of interest.  (c) Cohort study—summarise follow-up time (eg, average and total amount). | — | — | 11 |
| Outcome data | | | | |
| 15 | Cohort study—report numbers of outcome events or summary measures over time. Case-control study—report numbers in each exposure category, or summary measures of exposure. Cross sectional study—report numbers of outcome events or summary measures. | — | — | 13-14 |
| Main results | | | | |
| 16 | (a) Give unadjusted estimates and, if applicable, confounder adjusted estimates and their precision (eg, 95% confidence intervals). Make clear which confounders were adjusted for and why they were included.  (b) Report category boundaries when continuous variables are categorised.  (c) If relevant, consider translating estimates of relative risk into absolute risk for a meaningful time period. | — | — | 13-14 |
| Other analyses | | | | |
| 17 | Report other analyses done—eg, analyses of subgroups and interactions, and sensitivity analyses. | — | — | 10 |
| **Discussion** | | | | |
| Key results | | | | |
| 18 | Summarise key results with reference to study objectives. | — | — | 10-14 |
| Limitations | | | | |
| 19 | Discuss limitations of the study, taking into account sources of potential bias or imprecision. Discuss both direction and magnitude of any potential bias. | 19.1: Discuss the implications of using data that were not created or collected to answer the specific research question(s). Include discussion of misclassification bias, unmeasured confounding, missing data, and changing eligibility over time, as they pertain to the study being reported. | 19.1.a: Describe the degree to which the chosen database(s) adequately captures the drug exposure(s) of interest. | 17-18 |
| Interpretation | | | | |
| 20 | Give a cautious overall interpretation of results considering objectives, limitations, multiplicity of analyses, results from similar studies, and other relevant evidence. | — | 20.a: Discuss the potential for confounding by indication, contraindication or disease severity or selection bias (healthy adherer/sick stopper) as alternative explanations for the study findings when relevant. **[A: Original text indicated this item was RECORD (ie, not RECORD-PE)?]** | 16 |
| Generalisability | | | | |
| 21 | Discuss the generalisability (external validity) of the study results. | — | — | 17 |
| **Other information** | | | | |
| Funding | | | | |
| 22 | Give the source of funding and the role of the funders for the present study and, if applicable, for the original study on which the present article is based. | — | — | 19 |
| Accessibility of protocol, raw data, and programming code | | | | |
| 22 | — | 22.1: Authors should provide information on how to access any supplemental information such as the study protocol, raw data, or programming code. | — | 19 |

RECORD=reporting of studies conducted using observational routinely collected data; RECORD-PE=RECORD for pharmacoepidemiological research; STROBE=strengthening the reporting of observational studies in epidemiology.

*[REFERENCE: Langan SM, Schmidt S, Wing K, Ehrenstein V, Nicholls S, Filion K, Klungel O, Petersen I, Sorensen H, Guttmann A, Harron K, Hemkens L, Moher D, Schneeweiss S, Smeeth L, Sturkenboom M, von Elm E, Wang S, Benchimol EI. The REporting of studies Conducted using Observational Routinely-collected health Data (RECORD) Statement for Pharmacoepidemiology (RECORD-PE). *BMJ* 2018; 363: k3532.](https://www.bmj.com/content/363/bmj.k3532)

References

1. Ludvigsson JF, Almqvist C, Bonamy AKE, et al. Registers of the Swedish total population and their use in medical research. *Eur J Epidemiol*. 2016;31(2):125-136. doi:10.1007/s10654-016-0117-y

2. Brooke HL, Talbäck M, Hörnblad J, et al. The Swedish cause of death register. *Eur J Epidemiol*. 2017;32(9):765-773. doi:10.1007/s10654-017-0316-1

3. Ludvigsson JF, Andersson E, Ekbom A, et al. External review and validation of the Swedish national inpatient register. *BMC Public Health*. 2011;11. doi:10.1186/1471-2458-11-450

4. Wallerstedt SM, Wettermark B, Hoffmann M. The First Decade with the Swedish Prescribed Drug Register – A Systematic Review of the Output in the Scientific Literature. *Basic Clin Pharmacol Toxicol*. 2016;119(5):464-469. doi:10.1111/bcpt.12613

5. Wettermark B, Hammar N, Fored CM, et al. The new Swedish Prescribed Drug Register Opportunities for pharmacoepidemiological research and experience from the first six months. *Pharmacoepidemiol Drug Saf*. 2007;16(7):726-735. doi:10.1002/pds.1294

6. Karin H. The Swedish educational system and classifying education using the ISCED‐97. The International Standard of Classification of Education (ISCED-97).: An Evaluation of Content and Criterion Validity in 15 European Countries.

7. Morin L. *Too Much, Too Late? Drug Prescribing for Older People near the End of Life*.; 2019. https://ki.box.com/shared/static/zdtszjxbuf94i0qyk0fju7r2zsm4oc1f.pdf

8. Calderón-Larrañaga A, Vetrano DL, Onder G, et al. Assessing and Measuring Chronic Multimorbidity in the Older Population: A Proposal for Its Operationalization. *Journals of Gerontology - Series A Biological Sciences and Medical Sciences*. 2017;72(10):1417-1423. doi:10.1093/gerona/glw233
